# Community Interventions to Promote Mental Health and Social Equity

**DOI:** 10.1007/s11920-019-1017-0

**Published:** 2019-03-29

**Authors:** Enrico G. Castillo, Roya Ijadi-Maghsoodi, Sonya Shadravan, Elizabeth Moore, Michael O. Mensah, Mary Docherty, Maria Gabriela Aguilera Nunez, Nicolás Barcelo, Nichole Goodsmith, Laura E. Halpin, Isabella Morton, Joseph Mango, Alanna E. Montero, Sara Rahmanian Koushkaki, Elizabeth Bromley, Bowen Chung, Felica Jones, Sonya Gabrielian, Lillian Gelberg, Jared M. Greenberg, Ippolytos Kalofonos, Sheryl H. Kataoka, Jeanne Miranda, Harold A. Pincus, Bonnie T. Zima, Kenneth B. Wells

**Affiliations:** 1 Jane and Terry Semel Institute for Neuroscience and Human Behavior at UCLA, Department of Psychiatry and Biobehavioral Sciences, David Geffen School of Medicine, UCLA, Los Angeles, CA USA; 2 Center for Social Medicine and Humanities, UCLA, Los Angeles, CA USA; 3 Los Angeles County Department of Mental Health, Los Angeles, CA USA; 4 Division of Population Behavioral Health, Department of Psychiatry and Biobehavioral Sciences, David Geffen School of Medicine, UCLA, Los Angeles, CA USA; 5 VA Health Service Research and Development Center for the Study of Healthcare Innovation, Implementation, and Policy, VA Greater Los Angeles Healthcare System, Los Angeles, CA USA; 6 Harkness Fellow in Healthcare Policy and Practice, New York State Psychiatric Institute, Columbia University, New York, NY USA; 7 Center for Health Services and Society, UCLA, Los Angeles, CA USA; 8 UCLA Department of Anthropology, Los Angeles, CA USA; 9 Rand Corporation, Santa Monica, CA USA; 10 VA Greater Los Angeles Healthcare System, Los Angeles, CA USA; 11 Los Angeles Biomedical Research Institute, Los Angeles, CA USA; 12 Healthy African American Families II, Los Angeles, CA USA; 13 Department of Family Medicine, David Geffen School of Medicine, UCLA, Los Angeles, CA USA; 14 UCLA Jonathan Fielding School of Public Health, Los Angeles, CA USA; 15 UCLA International Institute, Los Angeles, CA USA; 16 Division of Child and Adolescent Psychiatry, UCLA, Los Angeles, CA USA; 17 Department of Psychiatry, Columbia University Medical Center, New York State Psychiatric Institute, NewYork-Presbyterian Hospital, Irving Institute for Clinical and Translational Research, New York, NY USA

**Keywords:** Mental health (MeSH), Mental health intervention (MeSH), Community networks (MeSH), Social problems (MeSH), Community interventions (MeSH), Community-based interventions (MeSH), Social determinants of health, Mental health equity, Health disparities, Multi-sector interventions

## Abstract

**Purpose of Review:**

We review recent community interventions to promote mental health and social equity. We define community interventions as those that involve multi-sector partnerships, emphasize community members as integral to the intervention, and/or deliver services in community settings. We examine literature in seven topic areas: collaborative care, early psychosis, school-based interventions, homelessness, criminal justice, global mental health, and mental health promotion/prevention. We adapt the social-ecological model for health promotion and provide a framework for understanding the actions of community interventions.

**Recent Findings:**

There are recent examples of effective interventions in each topic area. The majority of interventions focus on individual, family/interpersonal, and program/institutional social-ecological levels, with few intervening on whole communities or involving multiple non-healthcare sectors. Findings from many studies reinforce the interplay among mental health, interpersonal relationships, and social determinants of health.

**Summary:**

There is evidence for the effectiveness of community interventions for improving mental health and some social outcomes across social-ecological levels. Studies indicate the importance of ongoing resources and training to maintain long-term outcomes, explicit attention to ethics and processes to foster equitable partnerships, and policy reform to support sustainable healthcare-community collaborations.

**Electronic supplementary material:**

The online version of this article (10.1007/s11920-019-1017-0) contains supplementary material, which is available to authorized users.

## Introduction

Families, workplaces, schools, social services, institutions, and communities are potential resources to support health. In 1948, the World Health Organization defined health as a “state of complete physical, mental and social well-being and not merely the absence of disease or infirmity” [1]. Multi-sector and community-based mental healthcare approaches can help address health and social inequities by promoting social well-being and addressing structural determinants of mental health (public policies and other upstream forces that influence the social determinants of mental health).

A 2015 Cochrane review described three assumptions that underlie community interventions [2•]. The first is an awareness of the multiple forces that exist at all social-ecological levels (i.e., individual, interpersonal, organizational/institutional, community, and policy) that facilitate or obstruct mental health [3]. The second is investment in community participation to provide resources and inform interventions, recognizing expertise outside of the healthcare system. The third is prioritization of community mental health and social outcomes.

This review focuses on recent developments in community interventions to promote mental health. We highlight major developments and trends, rather than providing a comprehensive systematic review. Our review defines *community interventions* as those that involve multi-sector partnerships, include community members (e.g., lay health workers) as part of the intervention, and/or involve the delivery of services in community settings (e.g., schools, homes). We include interventions focused on traditional mental health outcomes (e.g., depression remission) and studies that include a wider range of outcomes including mental health-related knowledge, quality of life, and social well-being. We do not include substance use interventions, which warrant a separate review.

To complete our review, we enlisted a large team of experts and trainees with experience in pertinent intervention areas. Our review focuses on interventions published in peer-reviewed medical journals from 2015 to 2018, with additional studies identified through reference mining and expert recommendations. We concentrate on seven topic areas, chosen for their salience and quality of evidence in recent literature: multi-sector collaborative care, early psychosis interventions, school-based interventions, homeless services, criminal justice, global mental health, and mental health promotion and secondary prevention. We selected studies for their design, outcomes, and/or impact (Appendix A). These were chosen from a larger number of relevant community interventions (Appendix B).

## Multi-sector Collaborative Care

Collaborative care models in mental health have historical roots in the Chronic Care Model (CCM) of chronic disease management [4, 5••]. The CCM envisioned a combination of health system reforms and community-based resources to support the ability of healthcare settings to improve outcomes for those with chronic illnesses [4]. Many collaborative care studies, often for depression, have focused on incorporating mental health services to varying degrees within primary care settings [6–10]. Adaptations exist for other target populations (e.g., children) and settings (e.g., obstetrics/gynecology practices, mental health clinics) [5••, 11–13]. Studies have noted the importance of community organizations and social services, particularly when inequities play a large role in determining outcomes and require services beyond the healthcare sector, for example for underresourced populations and natural disasters [5••, 14, 15, 16, 17••].

Community Partners in Care (CPIC) was a depression collaborative care study that involved 95 programs in five sectors: outpatient primary care, outpatient mental health, substance use treatment services, homeless services, and other community services (e.g., senior centers, churches) [18•]. A 2015 Cochrane review identified CPIC as the only “high-quality study” that “specifically evaluated the added value of a community engagement and planning intervention (i.e. a coalition-led intervention) over and above resource enhancement and community outreach” [2•] (page 32). CPIC was a group-level randomized study that compared two program-level quality improvement interventions: Community Engagement and Planning (CEP) and Resources for Services (RS). RS programs received a depression care toolkit with technical assistance and consultation to implement a community-wide approach to depression care. CEP programs received the same resources within a multi-sector coalition approach to co-leading, implementing, and monitoring multi-sector depression services (e.g., encouraging community programs to be active in psychoeducation and screening, with streamlined referrals to clinics and social services) [19]. CPIC’s community-partnered participatory research approach and development of community partnerships are described in detail in several articles [19–24].

Unlike many collaborative care studies, CPIC focused on a predominantly under-resourced racial/ethnic minority sample (*n* = 1018, 46% African American, 41% Latino, 74% with family incomes below federal poverty level) and had few exclusion criteria, enrolling many participants with co-morbid substance use disorders and serious mental illnesses in the study [25, 26]. At 6-month follow-up, participants in CEP (*n* = 514) compared to RS (*n* = 504) had significantly improved health-related quality of life, increased physical activity, reduced homelessness risk factors, and reduced behavioral health hospitalizations [18•]. Sub-group analyses and follow-up studies at 12 and 36 months support some significant beneficial effects of CEP over RS, with main effects seen predominantly during the first 6 months post-intervention and diminishing over time [25, 27–34, 35•].

Since CPIC, only a handful of collaborative care studies have included non-healthcare partners [36–38, 39•]. Hankerson et al. conducted depression screenings in three predominantly African American Christian “mega churches” (≥ 2000 worshippers per weekend) in New York City, using a community coalition approach, including faith-based organizations and local government [38]. Investigators screened 122 community members at 3 church events in 2012. Notably, 19.7% of those screened reported moderate depression (PHQ-9 ≥ 10), in which the authors noted is higher than is seen in African American community samples. Moreover, none of the participants who screened positive requested community mental health referrals, even though these were offered, demonstrating the importance of churches as sites for depression screening, counseling (i.e., Mental Health First Aid), and referral [38, 39•].

## Early Intervention Services for Psychosis

There is a large and growing body of literature on coordinated specialty care programs for people with early psychosis, including the RAISE Early Treatment Program/NAVIGATE and OnTrackNY [40–47, 48•]. Germane to our community intervention focus, several early psychosis interventions summarized in a 2014 review by Nordentoft et al. adapted Assertive Community Treatment (ACT), an evidence-based service delivery model that emphasizes outreach-based services [48•, 49].

Secher et al. published the 10-year follow-up results of the Danish OPUS trial, a two-site RCT of a 2-year ACT-based assertive early intervention [50]. Services were delivered by a multidisciplinary team (psychiatrist, psychologists, nurses, social workers, vocational therapist, physiotherapist, 10:1 patient-to-staff ratio) in patients’ homes, other community locations, or clinic, based on patients’ preferences. Intensive services at this early critical stage were hypothesized to yield lasting effects by teaching individuals the skills to best manage their psychotic illnesses. OPUS results at 2 years showed significant positive outcomes compared to services as usual: decreased positive and negative psychotic symptoms, reduced substance use, improved treatment adherence, lower antipsychotic medication dosage, higher treatment satisfaction, and reduced family burden. At 10-year follow-up, however, most of these outcome differences had dissipated. Investigators conclude that longer duration of specialized assertive early intervention treatment, booster sessions, or the addition of an early detection program to reduce duration of untreated psychosis would aid the consolidation of early treatment gains.

An initiative by a London Early Intervention Service (EIS) sought to decrease duration of untreated psychosis and increase referrals from the community through early psychosis psychoeducational workshops with 36 community organizations (e.g., housing and social services, youth services, cultural and faith groups, police, colleges, employment agencies) [51•]. EIS staff conducted 41 half-day workshops at community organizations; monthly follow-up meetings and an additional session were offered; EIS promotional materials were made available; and EIS referral processes were streamlined for community organizations, including a linkage worker as a community liaison. Although the majority of community staff were in contact with people experiencing early psychosis in the past year (59.4%) and attitudes toward EIS as a first referral destination improved (37% pre- to 68% post-workshop), the study results were negative. Comparing EIS referrals in the year pre-/post-interventions, there was no significant difference in duration of untreated psychosis (295 vs. 396 days, *p* = 0.715) and, contrary to expectations, referred patients experienced significantly more contacts with intermediate healthcare/non-healthcare programs in their pathway to EIS treatment (2.06 vs. 2.45 steps, *p* = 0.002), reflecting a less streamlined referral process. In follow-up interviews, the authors note the barriers of mental health stigma, high community staff turnover, and resistance by EIS clinic staff to community-based work. Similar to CPIC, both of these studies suggest the importance of resources to sustain lasting change.

## School-Based Interventions

Research shows that youth, especially under-resourced youth, are most likely to receive mental healthcare in schools, given barriers to obtaining community mental health services [52••, 53]. School infrastructures also allow for large-scale implementation of prevention interventions [54••]. Given the number of factors involved in delivering school interventions, however, experts urge consideration of policies, school culture and climate, and leadership structure when delivering interventions [55, 56]. Academic outcomes can be difficult for researchers to collect given the unique requirements of Family Educational Rights and Privacy Act and HIPAA [57]. Further, developing sustainable interventions in schools that are truly responsive to the needs of students may require years of building academic-community partnerships [58].

Skryabina et al. assessed educational outcomes in an RCT of a universal school-based cognitive behavioral therapy prevention program, called FRIENDS [59]. FRIENDS is a manualized program that teaches emotional regulation, anxiety management, and problem solving, led by trained school staff or other designated health leaders. Forty-one schools were randomized to three arms (*n* = 1343): health-led FRIENDS, school-led FRIENDS, and a comparison group of Personal, Social, and Health Education (PSHE, emotional regulation, and self-awareness skills with less focus on anxiety management) which was provided by school staff. Health-led FRIENDS was more effective in decreasing social anxiety, generalized anxiety, and total Revised Children’s Anxiety and Depression Scale scores as compared to school-led FRIENDS and PSHE. There were no intervention effects on math, reading, or writing standardized assessment test scores.

Several studies implemented preventive interventions in the pre-kindergarten years. One such study evaluated developmental trajectories of youth, including behavioral, social, and learning measures over a 5-year period after receiving an enriched Head Start Curriculum [60]. This study is notable for its goal to address disparities and for the measures used to evaluate effects on development, which included social and learning behaviors and interpersonal relationships. In this RCT, 25 Head Start Centers were stratified and randomly assigned to receive usual Head Start vs. REDI intervention. REDI comprised dialogic reading, sound games, an interactive alphabet activity, and implementation of the Preschool Promoting Alternative Thinking Strategies curriculum focused on social emotional skills, with added professional development for teachers. Outcomes were obtained for 325 children who were followed for 5 years post-preschool. Children in the Head Start REDI intervention vs. control group were significantly more likely to follow optimal developmental trajectories in social behavior, aggressive-oppositional behavior, learning engagement, attention problems, student-teacher closeness, and peer rejection. This and other studies illustrate the importance of intervening at the levels of the classroom and whole school.

## Homeless Services

Individuals experiencing homelessness are at increased risk for mental illness, trauma, suicide, and medical comorbidities, along with a reduced life expectancy compared with the general population [61–64]. The recent focus on Housing First in community-based research on homelessness largely reflects an increasing embrace of that model [65]. Housing First is an approach to providing permanent housing without requirements for pre-placement sobriety or treatment participation [65]. Studies have demonstrated that Housing First yields quicker and more sustained housing retention compared to continuum housing approaches (transitional housing +/- sobriety or treatment requirements) [66••].

In the Canadian At Home/Chez Moi study, a multi-city RCT of the Housing First model compared with usual care, Aubry et al. followed 950 homeless or precariously housed adults with serious mental illness [67••]. The study found that participants in Housing First, compared with usual care, more quickly entered housing (within 73 vs. 220 days), retained housing for longer durations (281 vs. 115 days), and rated the quality of their housing more positively at 2-year follow-up. They also had significantly higher gains in community functioning and quality of life in the first year.

Several family-focused studies addressed homelessness. Nath examined the impact of drop-in homeless service centers for children in New Delhi, India [68]. They found that for every month of attendance at a drop-in center, children experienced 2.1% fewer ill health outcomes per month and used 4.6% fewer substances. Shinn et al. focused on social and mental health outcomes in children within newly homeless families with mental health or substance use disorders [69]. They compared usual care with a family-adapted critical time intervention, which combined housing and case management to connect families leaving shelters with community services. Youth in both groups exhibited reductions in psychosocial and mental health symptoms over time. Children ages 6–10 and 11–16 receiving the intervention compared to usual care were less likely at 24-month follow-up to self-report school troubles (i.e., suspension, being sent to the principal’s office, and being sent home with a note). Other studies have begun to analogously assess homeless interventions for broader social outcomes, including community functioning, arrests, public and other service use (e.g., food banks, shelters, prison time), employment, and income [70–74]. Future studies would benefit from expanded exploration of social outcomes that are important to individuals who have experienced homelessness.

## Criminal Justice

Nearly 40% of jail and prison inmates self-report a history of mental illness, and this prevalence is higher among those with more arrests and time served in a correctional facility [75]. Community interventions in collaboration with the criminal justice system are well positioned to address health disparities experienced by justice-involved populations and the vulnerabilities to justice involvement experienced by those with mental illness in the community. The studies below collaborated with the justice system to alter institutional (e.g., police, court) processes for those with mental illness and/or addressed upstream social and structural recidivism risk factors [76].

In Monroe County, New York, adults with psychotic disorders charged with misdemeanors were conditionally released and randomized to usual treatment (*n* = 35) or Forensic Assertive Community Treatment (FACT) (*n* = 35) [77]. FACT employed high-fidelity ACT services with the following adaptations: a 6-h training in criminal justice collaboration for clinicians, screening for criminogenic risk factors among enrollees, weekly court appearances, and meetings to discuss barriers to success with the supervising judge, public defender, and district attorney. Over a year, FACT enrollees had significantly fewer convictions (0.4 ± 0.7 vs 0.9 ± 1.3, *p* = .023), days in jail (21.5 ± 25.9 vs 43.5 ± 59.2, *p* = .025), and more days in outpatient mental health treatment (305.5 ± 92.1 versus 169.4 ± 139.6, *p* < .001) compared to treatment as usual.

A pilot study examined a social worker-administered decision-making intervention for police encountering people with mental illness [78•]. During the study period, any police officer who ran a background check on a detained enrollee was notified of enrollee participation in the program and was given the option to call a linkage specialist, usually a social worker employed by a community mental health agency. Linkage specialists provided mental health history (e.g., treatment participation, medication history) and treatment referral options. While this feasibility study lacked statistical power, the authors suggest that these results show the promise of a cross-sector approach to reducing arrests in this population.

Other interventions addressed risk factors for justice involvement like lack of insurance, unemployment, emotional regulation, and academic achievement [79–81, 82•, 83]. Two quasi-experimental studies focused on healthcare access, examining the downstream service use and recidivism effects of expedited Medicaid enrollment for recent prison releasees with schizophrenia or bipolar disorder in Washington State (*n* = 3086) [79, 80]. Twelve months post-implementation, 81% of the expedited group and 43% of the services as usual group were enrolled in Medicaid, (*p* < .01). Community mental health (69% vs. 37%, *p* < .01), outpatient primary care (64% vs. 42%, *p* < .01), and emergency room use (55% vs. 35%, *p* < .01) significantly increased in the intervention group compared to services as usual. Unexpectedly, there was a significantly greater proportion of those in the intervention versus comparison group that spent any days in jail (43 vs. 34%, *p* < .01) and state prison (56% vs. 46%, *p* < .01), with no significant difference in the proportion with any arrests (59% vs. 54%) at follow-up. The investigators suggest that while healthcare access is an important determinant for mental health, future interventions and policies must intentionally address the larger ecosystem of social/structural determinants of criminal justice involvement.

## Global Mental Health

Global mental health is “an area for study, research and practice that places a priority on improving health and achieving equity in health for all people worldwide” [84] (pg. 1995). We reviewed community interventions in international settings, acknowledging the shared social, structural, and mental health challenges that exist across nations. Many of the reviewed studies involve lay health worker (LHW) interventions [85•, 86–90]. Barnett et al. in their 2018 review of LHW interventions describe that LHWs elevate demand for services by increasing awareness of services and mental health literacy and by reducing stigma and barriers to care [85•]. Further, LHW interventions increase the supply of services in under-resourced areas by enlarging the workforce of culturally appropriate providers.

In 2017, Patel et al. published the first trial of a psychological intervention in primary care delivered by LHWs for moderate/severe depression in a low/middle income country [91•]. In that RCT, 495 participants in Goa, India, were assigned to the Healthy Activity Program (HAP) plus Enhanced Usual Care (EUC) intervention or EUC alone (usual care plus depression screenings and guideline-based primary care treatment of depression). In order to deliver the HAP (6–8 sessions on principles of behavioral activation), counselors received a 3-week training and 6-month internship under supervision of local mental health workers, who were trained by an expert on behavioral activation. At 3 months, HAP participants demonstrated significantly reduced depression symptom severity, suicidal ideation, disability, days out of work, and intimate partner violence and significantly higher rates of depression remission and improved behavioral activation compared to the EUC group.

A study in the Eastern Cape, South Africa, was the first to examine the effectiveness of a child abuse prevention program for adolescents in a low/middle income country [92••]. Most of the participating adolescents and caregivers (*n* = 115 dyads) from six under-resourced rural and peri-urban communities were referred to the study by non-governmental organizations, schools, clinics, chieftans, and social workers based on a history of family conflicts. Sixty percent of adolescent participants at baseline had either an HIV-positive caregiver or were orphaned by AIDS, 63% experienced pre-intervention child abuse, and 50% of caregivers at baseline endorsed intimate partner violence. Participants completed a 12-week parenting program delivered by local childcare workers. The study yielded significant improvements in social outcomes: reduced child abuse (63.0% to 29.5%, *p* < .001), reduced adolescent delinquency/aggressive behavior, reduced witnessed violence by adolescents, improved positive and involved parenting (adolescent and caregiver self-report), and improved social support (adolescent and caregiver self-report). The study also demonstrated significantly improved mental health outcomes, specifically decreased caregiver substance use, reduced adolescent and caregiver depression, and reduced parenting stress. These findings illustrate the interplay among social determinants, family dynamics, and caregiver-adolescent mental health.

Multiple recent studies consider the effects of war and broad structural forces on mental health [87–89, 93]. Cilliers et al. assessed the individual and community mental and social well-being outcomes associated with truth and reconciliation commissions (TRCs) in 200 Sierra Leone villages [94]. TRCs are community forums created to uncover wrongdoing by governments or other actors in the aftermath of major conflicts. The authors measured “societal healing” indicators, including forgiveness of perpetrators, trust, strength of social network, and community engagement, and “individual healing” indicators: PTSD, anxiety, and depression symptoms (*n* = 2383). They found that TRCs yielded improvements in societal healing, but worsened individuals’ health (worsened psychological health, depression, anxiety, and PTSD). The authors suggest policy implications such as integrated counseling in TRCs, reducing delays in holding TRCs after war, and exploring alternative post-conflict unification methods.

## Mental Health Promotion and Prevention

Communities That Care (CTC) is a community-level prevention planning and implementation system with primary foci on preventing youth (school grades 6–9) substance use, violence, and delinquency and secondary foci on depression, suicide, and other mental health outcomes. The CTC system involves five phases: identification of community stakeholders, formation of a community coalition, development of a community profile to identify risk and protective factors related to youth health and behavior problems, creation of a community action plan, and implementation and evaluation [95]. Communities implement evidence-based programs from the Building Healthy Youth Development registry, maintained by the University of Colorado Boulder’s Center for the Study of Prevention and Violence [96]. The Community Youth Development Study was a community-randomized study of CTC involving 24 communities (*n* > 14,000) in Colorado, Illinois, Kansas, Maine, Oregon, Utah, and Washington State [97–99]. CTC has also been implemented in Pennsylvania and rural Massachusetts [100–102]. In CTC versus control communities, results showed improved individual outcomes at eighth grade: reduced substance use, delinquency, and violence; later initiation of alcohol use, tobacco use, and delinquency; and lower prevalence of risky behaviors (past-year delinquency, past 2-week delinquency, and past-month alcohol and tobacco use) [103•]. Many of these results persisted to grades 10–12, despite few CTC programs focused on these grade levels. Fewer results (greater lifetime abstinence from antisocial behavior; greater lifetime abstinence from drug use and violence in male but not female participants) persisted to age 19 [103•, 104].

CTC investigators recently published follow-up results for participants at age 21 (*n* = 4002, 91% of the initial sample from grades 5–6), 11 years after initial CTC implementation [103•]. By age 21, CTC vs. control communities showed increased likelihood of lifetime abstinence from alcohol, tobacco, and marijuana use (ARR 1.49; 95% CI 1.03, 2.16), increased abstinence from antisocial behavior (ARR 1.18, 95% CI 1.02, 1.37), and decreased lifetime incidence of violence (ARR 0.89, 95% CI 0.79, 0.99). In male participants, CTC versus control communities also showed increased likelihood of sustained abstinence from tobacco, marijuana, and inhalant use.

Social protection studies investigate mental health and other outcomes associated with direct provision of resources in the forms of cash and food transfers [105, 106•, 107•, 108, 109]. A neighborhood cluster RCT in Ecuador investigated the effects of such resources on mental well-being and intimate partner violence [106•, 109]. Colombian refugees and low-income households in northern Ecuador were randomized to cash, food vouchers, food, or control arms. Treatment arms received the equivalent of $40 per month per household for 6 months, which represents 11% of pre-transfer monthly consumption. Food vouchers were redeemable at local supermarkets for a pre-approved list of nutritious foods. Food transfers were in the form of rice, lentils, vegetable oil, and canned sardines. Pooled results from all treatment arms showed the intervention significantly decreased the probability of controlling behaviors and physical and/or sexual violence by 6 to 7 percentage points compared to controls, with even greater reductions in the prevalence of any physical/sexual violence for women with low baseline ratings of household decision-making power [106•]. Qualitative interviews with participants indicated that improved family well-being, reduced marital stress and conflict, and women’s increased freedom of movement and decision-making power contributed to the decrease in violence. Similar studies include a large cluster RCT of cash transfers in Kenya’s program for at-risk youth and a cluster RCT of greening urban vacant land; both showed significant improvements in depression outcomes compared to control communities. These studies highlight the importance of addressing social inequities to achieve mental health gains in under-resourced communities [107•, 110•].

## Discussion

### Actions of Community Interventions by Social-Ecological Level

The community interventions above (Appendix A), drawn from a larger selection (Appendix B), highlight the successes and promise of these interventions to promote mental health and broader outcomes at all social-ecological levels: individual, interpersonal/family, organizational/institutional, community, and policy [3]. Community involvement is represented in varied ways in the form of individuals (lay health workers), settings (churches, schools), leaders (community-based participatory research), and multi-sector coalitions [35•, 37, 38, 39•, 85•, 86–90, 91•, 103•]. Many studies examined the interplay among mental health services, social and structural determinants, and mental health outcomes. Some explicitly assessed social outcomes like intimate partner violence, housing retention, academic performance, parent-child interactions, “societal healing,” and other contributors to mental and social well-being [67••, 92••, 94, 111].

**Fig. 1:**
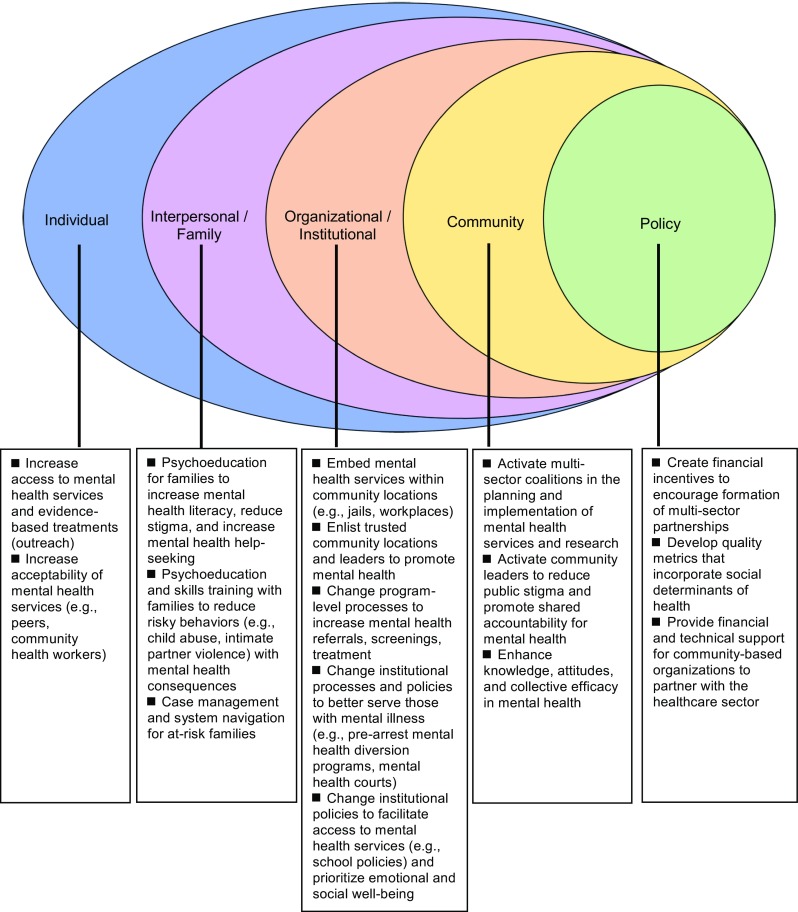
Overview of community intervention processes by social-ecological level (adapted from McElroy, KR, Bibeau D, Steckler A, Glanz K. An ecological perspective on health promotion programs. Health Educ Q. 1988;15:351–377)

Figure 1 summarizes the actions of community interventions by social-ecological level to promote mental health and social well-being. We found that most interventions reviewed promoted mental health at the individual level. LHW interventions extend access and increase acceptability of mental health services by leveraging trusted relationships. For example, Patel et al. demonstrated the successful delivery of behavioral activation for depression by LHWs through relatively brief training to a population with significant barriers to healthcare access [91•]. Some studies adapted evidence-based models (e.g., Forensic Assertive Community Treatment) to deliver treatments in non-traditional locations, such as jails, churches, and senior centers [77]. Many individual-level interventions also simultaneously acted at the organizational/institutional level. In the successful RCT of Head Start REDI, teachers were provided with professional development and mentoring to deliver an enriched curriculum [60].

A second group of interventions intervened at the interpersonal level (e.g., parent and family interventions). The effective child abuse prevention program in South Africa focused on the parent-child dyad through individual and joint sessions [92••]. Additionally, a strength of this intervention was its delivery by local child care workers. A third group of interventions functioned at the organizational/institutional level by enhancing the processes by which non-healthcare programs serve those with mental illness. These interventions enlisted non-healthcare entities and trusted community leaders to be active in mental healthcare, such as providing a depression screening intervention in churches [38, 39•]. Several successful school-based interventions operated at the organizational level, such as Warschburger and Zitzmann’s universal school-based prevention program for eating disorders in Germany and other whole school approaches [111, 112].

We found only a small number of studies that intervened at the level of whole communities. Most interventions reviewed here included one non-healthcare sector collaborator as opposed to collaborating with communities more broadly. Examples of community-level interventions include CPIC, which involved 95 organizations in 5 sectors to develop community-wide plans for managing depression, and CTC that supports communities to develop multi-sector coalitions to prevent youth substance use, violence, and delinquency [35•, 103•]. Other studies acted at the community level by directly providing or influencing resources on a large scale, through cash/food transfers or land revitalization efforts [94, 105, 106•, 107•, 108,109, 110•].

A fifth group of interventions are health and public policies. Policies that promote mental health equity are beyond the scope of this review but are detailed in our recent review on this topic [113•]. Policies as varied as mental health insurance parity, assisted outpatient treatment statutes, quality metrics for social determinants of health, value-based payment reforms, and the integration of funds and services for health and social care have the potential to improve access to treatment and improve outcomes [114–117, 118•, 119–121]. Policies facilitating multi-sector health collaborations include the Accountable Health Communities model, California’s Whole Person Care pilots, the Certified Community Behavioral Health Clinics Demonstration Program, New York’s Home and Community-based Services, the UK’s Social Impact Bonds Trailblazers, and the National Health Service England’s social prescribing teams [122–127]. Nation-level efforts to promote shared values for mental and social well-being are Australia’s mental health anti-stigma campaign, the US National Prevention Strategy’s focus on emotional well-being, and the UK’s Campaign to End Loneliness [128–130]. Thrive NYC is an example of large-scale action to promote mental health at the civic level, with a budget of $850 million and 54 initiatives across all public agencies and departments, with special emphases on community partnerships and prevention [131, 132•].

## Ethical Considerations

Ethical considerations are of importance to many community interventions given the focus on marginalized and under-resourced populations [24, 133]. Research on interventions for at-risk individuals with stigmatized conditions (e.g., incarceration, homelessness) should build trust with participants and recognize structural forces that place them at higher risk for these conditions (e.g., discriminatory policing and housing policies), to avoid inadvertently worsening stigma. Involving community stakeholders in equitable arrangements for interventions and research requires the necessary time and processes to develop effective partnerships. The expertise of community leaders and other stakeholders can be integrated equitably with that of researchers with trust, respect, and two-way knowledge exchange [134, 135]. Community-based organizations, social services, and healthcare agencies also have different funding streams and incentives. Efforts to sustain interventions should include a focus on funding and other enabling infrastructures (e.g., training, technology) for community groups to participate in intervention-related activities.

## Conclusions

There is evidence for the effectiveness of community interventions in multiple topic areas and acting at all social-ecological levels. International lay health worker interventions, a parenting intervention to reduce child abuse, a whole-school cognitive behavioral therapy prevention program, adapted ACT teams for early psychosis and justice-involved populations, Housing First services, and multi-sector collaborative care and prevention services are examples of effective community interventions. Studies indicate the importance of ongoing resources and training to maintain long-term outcomes and the need for policy reform to support healthcare-community partnerships. Future research should further define best practices for multi-sector collaborations and partnership structures, identify strategies for sustainable change after the end of research activities, and clarify the types of health and social problems that are best ameliorated through community interventions [2•]. In close and equitable partnerships with communities and policy leaders, future community interventions in mental health should seek to improve health and achieve large-scale social outcomes through initiatives that address mental health, structural, and social inequities.

## Electronic supplementary material

ESM 1 (DOCX 24 kb)

ESM 1 (DOCX 104 kb)
