# Supplementary material for: Community Interventions to Promote Mental Health and Social Equity
Source: Curr Psychiatry Rep. 2019 Mar 29;21(5):35. doi: 10.1007/s11920-019-1017-0 (PMC6440941; doi:10.1007/s11920-019-1017-0)
Supplement: Supplementary file 1 — (DOCX 24 kb) [file 11920_2019_1017_MOESM1_ESM.docx]

| **Table 1. Select Community Interventions** | | | | | | | | |
| --- | --- | --- | --- | --- | --- | --- | --- | --- |
| **Author** | **Title** | **Design** | **Interventions** | **Participants** | **Community Setting/ Involvement** | **Mental Health Outcomes** | **Individual or Community-level Social Outcomes** | **Other Outcomes** |
| Aubry et al. (2015) | One-year outcomes of a randomized controlled trial of housing first with ACT in five Canadian cities. | Nonblind, parallel-group Randomized controlled trial (RCT); outcomes over 1 year. | Housing First (rent supplement, assistance to find housing, and assertive community treatment) vs. treatment as usual. | n = 950 high-need participants with severe mental illness, who were either absolutely homeless or precariously housed in five Canadian cities (Vancouver, Winnipeg, Toronto, Montreal, and Moncton). | Health and social service agencies. | Improvement in overall quality of life was significantly greater among Housing First participants compared with treatment-as-usual participants. | At one-year follow-up, 73% of Housing First participants and 31% of treatment-as-usual participants resided in stable housing. Housing First participants also showed greater improvements in community functioning compared with treatment-as-usual participants. | None. |
| Cilliers et al. (2016) | Reconciling after civil conflict increases social capital but decreases individual well-being. | Randomized control trial; outcomes at 9 and 31 months. | Fambul Tok (community level reconciliation forums) treatment group vs. control group. | n = 2,383 individuals from 200 Senegalize villages. | 200 Senegaleze villages. | Worsened psychological health, increasing depression, anxiety, and posttraumatic stress disorder in these same villages. | Greater forgiveness of perpetrators and strengthened social capital: Social networks were larger, and people contributed more to public goods in treated villages. | None. |
| Cluver et al. (2016) | Reducing child abuse amongst adolescents in low- and middle-income countries: A pre-post trial in South Africa. | Pre-post study; outcomes at 2 to 6 weeks post intervention. | Parenting program facilitated by local NGO childcare workers for 12 weeks. | n = 230 participants, including youth and their caregivers in South Africa. No exclusion criteria. | Parenting program was held in local church halls or other public location in 6 rural and peri-urban communities in Eastern Cape South Africa; programming and research took place in close partnership with the Provincial and National Departments of Social Development and Education and UNICEF. | Reductions reported by adolescents and caregivers in adolescent aggressive behavior. Secondary outcomes showed reduced parental and adolescent depression and caregiver substance use. | Reductions reported by adolescents and caregivers in child abuse, poor monitoring/inconsistent discipline, adolescent deliquency, and improvements in positive/involved parenting. Secondary outcomes showed improved social support and reduced parenting stress. | None. |
| Compton et al. (2017) | A potential new form of jail diversion and reconnection to mental healthservices: II. Demonstration of feasibility. | Feasibility study; outcomes at 12 months. | Sequential Intercept Model Intercept 1, during the police-patient encounter, aiming to intervene at the pre‐booking diversion level. | n = 206 eligible individuals with serious mental illnesses and an arrest history. | Georgia's community service boards (CSBs), which are 26 quasi‐governmental mental health agencies that serve as the safety net for the provision of mental health services across the state. | Number of arrests decreased for individuals with serious mental illnesses. | Mental health services lack coordination and communication between the mental health and criminal justice systems. Here, researchers observed potential effectiveness of the linkage system in reducing the number of incarcerations in the year of enrollment in the system, as compared with the previous year. | None. |
| Hankerson et al. (2015) | Screening for Depression in African-American Churches. | Feasability study; outcomes on day of intervention. | Screen for depression in African American churches on a weeknight and featured a keynote address by the director of a national social service agency and panel discussion by mental health professionals. Treatment was not provided as part of the study. Survey was conducted at the program. | n = 122 participants from three predominantly African-American churches (two Baptist and one Methodist Episcopal) in New York City. Mean age was 53.7 years, and most were women (55.9%). 116 participants were black, 2 were Hispanic, 1 was Asian/Pacific Islander, and 2 were “other." | Three predominantly African-American churches involving key church leaders and researchers in New York City. Faith-Based Health Promotion (FBHP) program during the weekday and not during Sunday church service. | The prevalence estimate for positive depression screen was 19.7%. More men (22.5%) screened positive than women (17.7%). | Churches may be an important setting in which to identify depressive symptoms in this underserved population. Total household income was inversely related to positive depression screen. | This suggests that investigators should cultivate interdisciplinary relationships across clinical and community settings. |
| Hidrobo et al. (2016) | The effect of cash, vouchers, and food transfers on intimate partner violence: evidence from a randomized experiment in Northern Ecuador. | Neighborhood cluster randomized control study; outcomes at 6 months. | Six-month food assistance program made of four groups: cash, food transfers, food vouchers, and control. Participants attended monthly nutrition education sessions. | n = 80 neighborhoods and 145 clusters within neighborhoods; n = 2,357 households. | Seven urban centers in the northern provinces of Carchi and Sucumbíos consisting of Colombian refugees and low-income Ecuadorians households in Ecuador. | None. | Food transfers decrease the probability that a woman experiences controlling behaviors, and physical and/or sexual violence by 6 to 7 percentage points or approximately a 19 percent to 30 percent decrease from endline control means. And, there was no evidence that partners use violence to forcefully extract transfers. | None. |
| Kilburn et al. (2016) | Effects of a large-scale unconditional cash transfer program on mental health outcomes of young people in Kenya. | Experiment; outcomes over 4 years. | Monthly cash payments of $20 to households that are poor and have at least one orphan or vulnerable child below 18 years of age vs. control. | n = 1,960 households in the Cash Transfer for Orphans and Vulnerable Children (CT-OVC) prgram. | Households in the CT-OVC program in Kenya. | Young men living in households that received unconditional cash transfers were less likely to show depressive symptoms, more likely be hopeful about their lives, and more likely to be healthier than they were previously. The positive impact of the program is stronger among the subgroup of orphans. This study provides evidence that poverty-targeted unconditional cash transfer programs, can improve the mental health of young people in low-income countries. | For young men, residing in an intervention household increased the likelihood of feeling healthier from a year ago. Among young women, the effects of the CT-OVC program were not statistically significant. Finally, intervention effects on being healthy in the past 4 weeks were not statistically significant for young men or young women. | None. |
| Lamberti et al. (2017) | A Randomized Controlled Trial of the Rochester Forensic Assertive Community Treatment Model. | Randomized Controlled Trial; outcomes over 1 year. | Sequential Intercept Model Intercept 3, post-sentencing conditional discharge; forensic assertive community treatment (FACT) vs. enhanced treatment as usual for one year. | n = 70 adults with psychotic disorders who were arrested for misdemeanor crimes and who were eligible for conditional discharge were recruited from the Monroe County, New York, court system. | Care delivered in community locations in accordance with Assertive Community Treatment model of care; weekly meetings between a FACT team liaison, the presiding judge, and representatives from the Public Defender and District Attorney offices. | More time in outpatient mental health and less time in the hospitals. | Rochester FACT model was associated with fewer convictions for new crimes, less time in jail and hospitals, among justice-involved adults with psychotic disorders compared with treatment as usual. | None. |
| Lloyd-Evans et al. (2015) | Evaluation of a community awareness programme to reduce delays in referrals to early intervention services and enhance early detection of psychosis. | Quasi experimental- pre and post intervention questionnaires and mixed methods including qualitative analysis; outcomes at one year post intervention. | A one-year community awareness program about psychosis targeting staff in non-health service community organizations. The program comprised psycho-educational workshops and Early Intervention Services (EIS) link workers, and offering direct referral routes to EIS. | n = 367 staff over 41 workshops from non-health service community-based organizations in London, UK. | Awareness program delivered within the worplace of non-health community-based organizations involved with young people including youth and faith groups, employment, education and housing organisations, black and minority ethnic community groups, probation and social services, and the police in London, UK. | Participants’ knowledge and attitudes to psychosis and attitudes to mental health services improved significantly following workshops. | The community awareness program did not reduce treatment delays for people experiencing first episode psychosis. Further research is needed regarding effective means to reduce duration of untreated psychosis. | None. |
| Morrissey et al. (2016) | Expedited Medicaid Enrollment, Mental Health Service Use, and Criminal Recidivism Among Released Prisoners With Severe Mental Illness. | Quasi-experiment with linked administrative data; outcomes over 12 months. | Sequential Intercept Model Intercept 4; prison releasees referred to expedited Medicaid enrollment vs. control group (not referred). | n = 3,086 released prisoners with a severe mental illness (schizophrenia or bipolar disorder) in Washington State. | Washington State state prisons, expedited Medicaid program, and Community Service Officers. The linked administrative data was provided from the Washington State Department of Social and Health Services (DSHS) in the state of Washington. | Referral for expedited Medicaid enrollment on release from prison greatly increased Medicaid enrollment (p<.01) and use of community mental health and general medical services (p<.01) for persons with severe mental illness. | No evidence was found that expediting Medicaid enrollment reduced criminal recidivism. | None. |
| Nath et al. (2016) | The Impact of Drop-In Centres on the Health of Street Children in New Delhi, India. | Cross-sectional study; outcomes at 12 months. | Visits to one of two drop-in centers. | n = 134 homeless children in New Delhi, India (69 attenders and 65 nonattenders of drop-in centers). | Drop-in homeless service centers. | Attendees had better mental health outcomes than non-attendees. | None. | For every month of attendance at a drop-in center, children experienced 2.1% fewer ill health outcomes per month and used 4.6% fewer substances. Children were also less likely to have been a current substance user than a never substance user for every additional month of attendance at a center. |
| Nix et al. (2016) | The randomized controlled trial of Head Start REDI: Sustained effects on developmental trajectories of social-emotional functioning. | Randomized controlled trial; outcomes at end of intervention (1 year) and then annually for a further 4 years. | The 'Head Start REDI' intervention (Research-based, Developmentally-Informed). A one year enriched preschool curriculum involving integrated language, emergent literacy and social-emotional skills and enhanced support for positive teaching practices vs. 'Head Start' as usual. | n = 356 children recruited in final year of pre-school who attended Head Start in three Pennsylvania counties. 70% of the children came from families living in poverty. | Intervention delivered by teachers within primary school setting in the USA. | Impact of sustained effects on children’s developmental trajectories of social-emotional functioning four years after participation were measured using multiple scales. Children in the intervention group were statistically significantly more likely to exhibit the most optimal developmental trajectories of social competence, aggressive-oppositional behavior, learning engagement, attention problems, student-teacher closeness, and peer rejection. | None. | None. |
| Oesterle et al. (2018) | Long-Term Effects of the Communities That Care Trial on Substance Use, Antisocial Behavior, and Violence Through Age 21 Years. | Community-Randomized trial; outcomes at 11 years. | Communities That Care (CTC) prevention system, implemented in early adolescence to promote positive youth development and reduce health-risking behavior vs. control (communities given youth surveys but no funding or training from the study). | n = 24 matched communities/ towns in seven states in the USA; 4407 participants (grade 5 through age 21 years). Data for this study came from the Community Youth Development Study (CYDS) (Hawkins et al. 2008b), a community-randomized trial of CTC. | Community settings in Colorado, Illinois, Kansas, Maine, Oregon, Utah, and Washington State. | There was no significant overall effect of CTC on mental health secondary outcomes, including, major depression, and suicidality. | The CTC system increased the likelihood of sustained abstinence from gateway drug use by 49% and antisocial behavior by 18%, and reduced lifetime incidence of violence by 11% through age 21 years. In male participants, the CTC system also increased the likelihood of sustained abstinence from tobacco use by 30% and marijuana use by 24%, and reduced lifetime incidence of inhalant use by 18%. | This study provides evidence that the CTC is an effective approach to improving public health in the long term by preventing the incidence of health-risking behaviors many years after the most direct exposure to evidence-based programs and policies. |
| Ong et al. (2017) | A Community-Partnered, Participatory, Cluster-Randomized Study of Depression Care Quality Improvement: Three-Year Outcomes. | Cluster-randomized study; outcomes at 3 years. | Community Engagement and Planning (CEP) vs. individual program technical assistance (Resources for Services [RS]) for implementing depression quality improvement in underserved communities. | n = 600 depressed clients. Data from this study came from Community Partners in Care (CPIC) in South Los Angeles and Hollywood-Metro Los Angeles. | Health-care and community based agencies offering services identified by community members as relevant to depression (mental health specialty services, primary care, public health services, substance abuse treatment services, social services, faith-based services, park community centers, hair salons, and exercise clubs). | At three years, CEP and RS did not have differential effects on primary mental health outcomes. | At three years, CEP participants had modest effects in improving physical health-related quality of life and reducing behavioral health hospital nights. | None. |
| Patel et al. (2017) | The Healthy Activity Program (HAP), a lay counsellor-delivered brief psychological treatment for severe depression, in primary care in India: a randomised controlled trial. | Randomized controlled trial; outcomes at 3 months. | Healthy Activity Program (HAP): lay counselors conducted 6-8 sessions (behavioral activation as the core psychological framework with added emphasis on strategies such as problem-solving and activation of social networks) vs. enhanced usual care delivered by lay counsellors for 3 months. | n = 495 participants screening more than 14 on PHQ9 in India. | 10 primary care centers in Goa, India. | Intervention group had strong effect on depression remission and depression symptom severity at 3 months. Secondary outcomes also showed improvement in intervention group (disability score, days unable to work, behavioral activation score, SI, intimate partner physical violence experienced by women) however there was no change in intimate partner psychological/emotional violence experienced by men or women or intimate partner physical violence experienced by men. | Days unable to work in past 1 month are lower in the intervention group. Women who received HAP were nearly 50% less likely to report intimate partner physical violence at the end of treatment than were women in usual care. | None. |
| Secher et al. (2015) | Ten-year follow-up of the OPUS specialized early intervention trial for patients with a first episode of psychosis. | Randomized controlled trial; outcomes at 10 years post intervention. | Treatment as usual (TAU) (indefinitely) vs. 2 years of OPUS treatment followed by TAU (indefinitely). | n = 547 patients with a first episode of psychosis in Denmark. | Social workers, psychologists, psychiatric nurses, occupational therapists, and a psychiatrist as well as community mental health centers. | Of the 547 participants included in the study, 347 (63.4%) took part in this follow-up. While there was evidence of a differential 10-year course in the development of negative symptoms, psychiatric bed days, and possibly psychotic symptoms in favor of OPUS treatment, differences were driven by effects at earlier follow-ups and had diminished over time. | Statistically significant differences in the course of use of supported housing were present even after 8-10 years. There were no differences between OPUS and TAU regarding income, work-related outcomes, or marital status. | To further investigate the positive effects of OPUS, researchers suggest OPUS treatment should be extended to a period of 5 years. |
| Shinn et al. (2015) | Longitudinal Impact of a Family Critical Time Intervention on Children in High-Risk Families Experiencing Homelessness: A Randomized Trial. | Randomized trial; outcomes over 3, 9, 15, and 24 months. | FCTI (combines housing and structured, time-limited case management to connect families leaving shelter with community services) vs. usual care. | Children in 200 newly homeless families in which mothers had diagnosable mental illness or substance problems in Westchest County, NY. | Homeless shelter system. | Both experimental and control children in all age groups showed reductions in symptoms over time (mental health, psychosocial symptomjs). Although experimental results were scattered, they suggest that FCTI has the potential to improve mental health and school outcomes for children experiencing homelessness. | The intervention led to declines in self-reported school troubles for children aged 6-10 and 11-16. | None. |
| Skryabina et al. (2016) | Effect of a universal anxiety prevention programme (FRIENDS) on children's academic performance: results from a randomised controlled trial. | Randomized controlled trial; outcomes at 12 months post intervention. | Universal school-based cognitive behaviour therapy prevention program, FRIENDS, delivered by health care staff or school staff vs. usual personal, social, health and education (PSHE) lessons. | n = 1,343 primary school children in England. | Schools in England involving school staff or care staff along with intervention curriculum. | Found a singificant reduction in self-reported anxiety (social anxiety, GAD, total anxiety) when FRIENDS delivered by health leaders external to schools. | No effect found on academic perforamance of children 12 months after participating. | None. |
