# Supplementary material for: Community Interventions to Promote Mental Health and Social Equity
Source: Curr Psychiatry Rep. 2019 Mar 29;21(5):35. doi: 10.1007/s11920-019-1017-0 (PMC6440941; doi:10.1007/s11920-019-1017-0)
Supplement: Supplementary file 2 — (DOCX 104 kb) [file 11920_2019_1017_MOESM2_ESM.docx]

| **Community Interventions: Multi-sector Collaborative Care** | | | | |  |  |  |  |
| --- | --- | --- | --- | --- | --- | --- | --- | --- |
| **Author** | **Title** | **Design** | **Interventions** | **Participants** | **Community Setting/Involvement** | **Mental Health Outcomes** | **Individual or Community-level Social Outcomes** | **Other Outcomes** |
| Castillo et al. (2017) | Improving Depression Care for Adults With Serious Mental Illness in Underresourced Areas: Community Coalitions Versus Technical Support. | Secondary analysis of a group-randomized study; outcomes at 6 and 12 months. | Collaborative depression care delivered through Community Engagement and Planning (CEP), a coalition approach vs. Resources for Services (RS), a technical assistance approach. | n = 504 participants from racial-ethnic minority groups (50% had serious mental illness). Data from this study came from Community Partners in Care (CPIC) in South Los Angeles and Hollywood-Metro Los Angeles. | Programs from health care (primary care, public health, mental health, and substance abuse) and social-community sectors (homeless and social services, faith-based, park community centers, hair salons, exercise or other program). | CEP relative to RS reduced the likelihood of poor mental health–related quality of life but not depression (primary outcomes); increased the likelihood of mental wellness at six months. There were no statistically significant 12-month effects. | CEP relative to RS reduced the likelihood of having homelessness risk factors and behavioral health hospitalizations; reduced specialty mental health medication and counseling visits; and increased faith-based depression visits (each p<.05) at six months. There were no statistically significant 12-month effects. | None. |
| Choi et al. (2018) | A Comparative Effectiveness Trial of Depression Collaborative Care: Subanalysis of Comorbid Anxiety. | Cluster-randomized comparative-effectiveness trial; outcomes at 6 and 12 months. | Quality improvement materials, plus technical support (Resources for Services, RS) vs. Quality improvement materials, plus support for collaborative implementation planning (Community Engagement and Planning, CEP). | n = 1,018 participants (depression = 360; comorbid depression/anxiety = 658). Data from this study came from Community Partners in Care (CPIC) in South Los Angeles and Hollywood-Metro Los Angeles. | Two low-resource communities provided mental health care, primary care, substance use services, social services (homeless-serving, prisoner re-entry, or family preservation) or community-based services (faith-based, park and recreation community or senior centers, hair salons). | For the comorbid depression/anxiety subgroup, the collaborative planning arm was superior at improving mental health-related quality of life and mental wellness, at 6 months. The effects were not significant at 12 months. | For the comorbid depression/anxiety subgroup, the collaborative planning arm decreased behavioral hospitalizations and homelessness risk. The effects were not significant at 12 months. | None. |
| Chung et al. (2015) | Participation in Training for Depression Care Quality Improvement: A Randomized Trial of Community Engagement or Technical Support. | Matched programs study; outcomes over 12-16 weeks. | Resources for Services (RS) relied on providing more traditional technical assistance to individual programs vs. Community Engagement and Planning (CEP) used community-partnered, participatory research (CPPR) principles to support collaborative planning by a network of agencies seeking to implement the same depression care tool kits. | n = 1,622 staff members from social services and health care sectors | Social services and health care sectors in South Los Angeles and Hollywood-Metro Los Angeles. | CEP programs were more likely than RS programs to participate in any training (p=.006). Within health care sectors, CEP programs were more likely than RS programs to participate in training (p=.016), but within social-community sectors, there was no difference in training by intervention. | Study focused on program participation in training, not patient outcomes | None. |
| Chung et al. (2018) | 12-Month Cost Outcomes of Community Engagement Versus Technical Assistance for Depression Quality Improvement: A Partnered, Cluster Randomized, Comparative-Effectiveness Trial. | Cluster-randomized comparative-effectiveness trial; outcomes at 6 and 12 months. | Community engagement and planning (CEP) vs. resources for services (RS) for depression quality improvement. | n = 1013 adults with depressive symptoms (85% African American and Latino) in South Los Angeles and Hollywood-Metro Los Angeles. | Under-resourced communities, individual health care and community-based programs. | There were no significant differences by intervention status in participant characteristics at baseline. | CEP planning and training costs were almost 3 times higher than RS, largely due to greater CEP provider training participation vs. RS, with no significant differences in 12-month service-use costs. | None. |
| Grote et al. (2015) | Collaborative Care for Perinatal Depression in Socio-economically Disadvantaged Women: A Randomized Trial. | Randomized controlled trial; outcomes at 3, 6, 12, and 18 months post intervention. | “MOMCare,” a culturally relevant, collaborative care intervention, providing a choice of brief interpersonal psychotherapy and/or antidepressants vs. usual public health care Maternity Support Services (MSS). | n = 168 pregnant women on medicaid with probable depression or dysthymia in Seattle-King County Public Health System. | 10 public health system centers consisting of nutritionist, obstetrician, and community social worker provider team. | MOMCare (n = 83) compared to MSS-Plus participants (n = 85) attained significantly lower levels of depression severity and PTSD severity, higher rates of depression remission, and had a greater likelihood of receiving ≥4 mental health visits and of adhering to antidepressants in the prior month. | None. | None. |
| Hankerson et al. (2015) | Screening for Depression in African-American Churches. | Feasability study; outcomes on day of intervention. | Screen for depression in African American churches on a weeknight and featured a keynote address by the director of a national social service agency and panel discussion by mental health professionals. Treatment was not provided as part of the study. Survey was conducted at the program. | n = 122 participants from three predominantly African-American churches (two Baptist and one Methodist Episcopal) in New York City. Mean age was 53.7 years, and most were women (55.9%). 116 participants were black, 2 were Hispanic, 1 was Asian/Pacific Islander, and 2 were “other." | Three predominantly African-American churches involving key church leaders and researchers in New York City. Faith-Based Health Promotion (FBHP) program during the weekday and not during Sunday church service. | The prevalence estimate for positive depression screen was 19.7%. More men (22.5%) screened positive than women (17.7%). | Churches may be an important setting in which to identify depressive symptoms in this underserved population. Total household income was inversely related to positive depression screen. | This suggests that investigators should cultivate interdisciplinary relationships across clinical and community settings. |
| Hankerson et al. (2018) | Partnering with African American Churches to Create a Community Coalition for Mental Health. | Qualitative study; outcomes at 18 months. | Bi-monthly meetings with clergy for the first six months and quarterly thereafter. As well as a community mental health symposium. Training for evidence-based practices to African American faith leaders in Harlem, New York from two churches. | n = 14 members of the Community Coalition for Mental Health in Central Harlem, New York in a borough of Manhattan. | Two churches: Church #1 is a Baptist church with more than 8,000 members (98% African American); Church #2 is a Catholic parish with approximately 800 members (90% African American). | None. | The outcomes of the community-partnered Vision stage included creating a Community Coalition, establishing the group’s values, forming two subcommittees, and collaboratively deciding to change the study intervention to Mental Health First Aid (MHFA). | Support from the lead pastor is crucial; balancing community and academic interests can be challenging. |
| Henderson et al. (2017) | Integrated collaborative care teams to enhance service delivery to youth with mental health and substance use challenges: protocol for a pragmatic randomised controlled trial. | Randomized controlled trial; outcomes at 6 and 12 months. | Treatment as usual (TAU) at one of four outpatient hospital sites vs. Integrated Collaborative Care Team (ICCT) treatment at one of three community-based sites; ICCT is described as combining low, moderate and high-intensity services in a youth-friendly space; services include individual and group mental health services, DBT and CBT services, primary care, social activities, assertive outreach, and other services. | n = 500 youth presenting for hospital-based, outpatient psychiatric service in Toronto, Canada. | Outpatient hospital sites and ICCTs, which are are housed in the community in three neighbourhoods across Toronto, Canada; community-based sites; project is governed by multistakeholder groups, including representatives of the four hospital sites, community agencies, youth and family members. | The primary outcome variable will be the youth's functioning. | Secondary outcomes will include clinical change, youth/family satisfaction and perception of care, empowerment, engagement and the incremental cost-effectiveness ratio (ICER). | None. |
| Izquierdo et al. (2018) | Community Partners in Care: 6- and 12-month Outcomes of Community Engagement versus Technical Assistance to Implement Depression Collaborative Care among Depressed Older Adults. | Community-partnered, cluster-randomized trial; outcomes at 6 and 12 months. | A community-partnered multi-sector coalition approach (Community Engagement and Planning [CEP]) vs. individual program technical assistance (Resources for Services [RS]) to implement depression collaborative care. | n = 394 participants aged >50 years with depressive symptoms. Data from this study came from Community Partners in Care (CPIC) in South Los Angeles and Hollywood-Metro Los Angeles. | Service sectors (ie, settings) that support vulnerable depressed populations (low rates of insurance and high rates of poverty and avoidable hospitalizations). | At 6 months, CEP was more effective than RS at improving mental health-related quality of life (MHRQL) and mental wellness among participants aged >50 years. No significant outcome differences between CEP and RS were found at 12 months. | No differences were found in the effects of CEP vs. RS on Depressive symptoms (PHQ-8 score), community-prioritized outcomes including homelessness risk, and physical activity, and services utilization outcomes. No significant outcome differences between CEP and RS were found at 12 months. | None. |
| Lam et al. (2016) | The Impact of Community Engagement on Health, Social, and Utilization Outcomes in Depressed, Impoverished Populations: Secondary Findings from a Randomized Trial. | Randomized trial of matched health-sector and community-based programs; outcomes over 12 months. | Community Engagement and Planning (CEP) intervention cultivating multi-agency networks to implement depression quality improvement interventions across health and community sectors as a community coalition vs. Resources for Services (RS) made expert assistance available to individual agencies. | n = 1,018 adults living above (n = 268) and below (n=750) the federal poverty level; the latter group consisted of 3 subgroups: justice-involved (n=158), homeless and not justice-involved (n= 298), and other poor (n = 294) in two Los Angeles (LA) communities (South LA, Hollywood Metro). | Community coalition model entailing community input and coordination among health sector and community-based programs (e.g., primary care, mental health, homeless services, prison reentry services, faith-based programs, community centers, hair salons, exercise clubs). | CEP improved mental wellness among people with depression living below the federal poverty level. | For justice-involved participants at 6 months, CEP showed suggestive evidence of reducing the percentage currently homeless or having multiple homelessness risk factors, and of reducing total life difficulties. No significant findings for social outcomes within the homeless not justice-involved or "other poor" subgroups. | The development of a community-coalition across healthcare and community-based service programs coupled with training in approaches to screen, refer, support coping and deliver services to depressed clients, may have helped program staff alleviate stressors (e.g. food, shelter, clothing) or avoid arrest, for a population (depressed) often difficult to engage in services. |
| Landry et al. (2016) | The Effects of Collaborative Care Training on Case Managers' Perceived Depression-Related Services Delivery. | Cluster-randomized trial; outcomes at 1 year. | Community Engagement and Planning (CEP), which supports collaboration across health and community-based agencies, vs. Resources for Services (RS), which provides technical assistance, on training participation and service delivery by primarily unlicensed, racially and ethnically diverse case managers. | n = 117 case managers (N=59, RS; N=58, CEP) in two low-income communities. Data from this study came from Community Partners in Care (CPIC) in South Los Angeles and Hollywood-Metro Los Angeles. | 84 health and community-based programs. | Personal depression stigma was positively associated with depression case management. | CEP case managers had greater participation in depression training, spent more time providing services in community settings, and used more problem-solving therapeutic approaches compared with RS case managers. CEP offers a model for including case managers in communitywide depression care improvement efforts. | None. |
| Mehta et al. (2017) | Community Partners in Care: 6-Month Outcomes of Two Quality Improvement Depression Care Interventions in Male Participants. | Community partnered, cluster, randomized trial; outcomes at 6 months. | Depression collaborative care implementation using community engagement and planning (CEP) across programs vs. the more-traditional individual program, technical assistance (Resources for Services, RS). | n = 423 adult male clients with modified depression. Data from this study came from Community Partners in Care (CPIC) in South Los Angeles and Hollywood-Metro Los Angeles. | Health care and social-community sectors. | Intervention effects on primary outcomes (depressive symptoms (PHQ-8 score) and mental health-related quality of life) did not differ. Men in CEP compared with RS had improved mental wellness. | Men in CEP compared with RS had reduced hospitalizations, with fewer mental health specialty medication visits, and a trend toward greater faith-based depression visits. | None. |
| Ngo et al. (2016) | Community Engagement Compared With Technical Assistance to Disseminate Depression Care Among Low-Income, Minority Women: A Randomized Controlled Effectiveness Study. | Secondary analysis; matched, clustered, randomized trial; outcomes at 6 and 12 months. | Community Engagement and Planning (CEP) vs. a technical assistance approach (Resources for Services, or RS) to disseminate depression care. | n = 595 low-income, minority women in two low-resource communities. Data from this study came from Community Partners in Care (CPIC) in South Los Angeles and Hollywood-Metro Los Angeles. | Agencies and venues offering services identified by community members as relevant to depression (mental health specialty, primary care and public health, substance abuse, social services, faith-based services, park centers, hair salons, and exercise clubs). | There were statistically significant effects for mental health quality of life, and resiliency at 6 months. | There were statistically significant effects for homelessness risk, and financial difficulties at 6 months, as well as missed work days, self-efficacy, and care barriers at 12 months favoring CEP relative to RS. CEP increased use of outpatient substance abuse services and faith-based depression visits at 6 months. | The CPIC study results may be relevant to existing policy initiatives such as Medicaid Behavioral Health Homes and Accountable Care Communities, which incentivize health care and community agency partnerships to improve population-based health outcomes. |
| Ong et al. (2017) | A Community-Partnered, Participatory, Cluster-Randomized Study of Depression Care Quality Improvement: Three-Year Outcomes. | Cluster-randomized study; outcomes at 3 years. | Community Engagement and Planning (CEP) vs. individual program technical assistance (Resources for Services [RS]) for implementing depression quality improvement in underserved communities. | n = 600 depressed clients. Data from this study came from Community Partners in Care (CPIC) in South Los Angeles and Hollywood-Metro Los Angeles. | Health-care and community based agencies offering services identified by community members as relevant to depression (mental health specialty services, primary care, public health services, substance abuse treatment services, social services, faith-based services, park community centers, hair salons, and exercise clubs). | At three years, CEP and RS did not have differential effects on primary mental health outcomes. | At three years, CEP participants had modest effects in improving physical health-related quality of life and reducing behavioral health hospital nights. | None. |
| Sherbourne et al. (2017) | Comparative Effectiveness of Two Models of Depression Services Quality Improvement in Health and Community Sectors. | Comparative effectiveness trial; outcomes at 6, 12, or 36 months. | Community Engagement and Planning (CEP) vs. program technical assistance (Resources for Services) for implementing depression services quality improvement in underserved communities. | n = 1,018 participants from racial-ethnic minority groups. Data from this study came from Community Partners in Care (CPIC) in South Los Angeles and Hollywood-Metro Los Angeles. | Programs from health care (primary care, public health, mental health, and substance abuse programs) and community sectors (homelessness and social services, faith-based services, hair salons, park senior centers, and exercise clubs). | Compared with RS, CEP was associated with a higher likelihood of mental wellness at six months and greater PHRQL at 36 months among health care clients and with less homelessness risk at six months among social-community clients. | For services use, at 36 months, increases were found for coalition building in primary care visits, self-help visits, and appropriate treatment for community clients and in community-based services use for health care clients. | None. |
| Springgate et al. (2018) | Comparative Effectiveness of Coalitions Versus Technical Assistance for Depression Quality Improvement in Persons with Multiple Chronic Conditions. | Secondary analyses of a cluster-randomized trial; outcomes at 6 and 12 months. | Community Engagement and Planning (CEP) vs. Resources for Services (RS) for implementing depression quality improvement programs. | n = 1,018 adults with depressive symptoms. Data from this study came from Community Partners in Care (CPIC) in South Los Angeles and Hollywood-Metro Los Angeles. | Programs from health care (primary care, public health, mental health, and substance abuse) and social-community sectors (homeless and social services, faith-based, park community centers, hair salons, exercise or other program). | CEP relative to RS was associated with greater improvements at 6 months among multiple chronic medical condi­tions (MCC) participants in both primary outcomes of mental health-related quality of life (MHRQL) and depression. | Significant effects of CEP over RS for at least one time point among MCC participants for community-prioritized outcomes, ie, mental wellness, homelessness risk factors, ≥ 4 behavioral health hospitalizations, and work-loss days among employed. | Relative to those without MCC, participants with MCC were older, in worse mental and physical health, less likely to be working for pay. |

| **Community Interventions: Early Psychosis Interventions** | | | | |  |  |  |  |
| --- | --- | --- | --- | --- | --- | --- | --- | --- |
| **Author** | **Title** | **Design** | **Interventions** | **Participants** | **Community Setting/Involvement** | **Mental Health Outcomes** | **Individual or Community-level Social Outcomes** | **Other Outcomes** |
| Albert et al. (2017) | Five years of specialised early intervention versus two years of specialised early intervention followed by three years of standard treatment for patients with a first episode psychosis: randomised, superiority, parallel group trial in Denmark (OPUS II). | Randomized, superiority, parallel group trial; outcomes at 2 and 5 years. | OPUS treatment consists of three core elements—modified assertive community treatment, family involvement, and social skill training—with a patient-case manager ratio of no more than 12:1 vs. participants referred to community health centres after two years of specialised early intervention (SEI) treatment. | n = 400 participants with schizophrenia spectrum disorders in the OPUS treatment program in Denmark. | Six OPUS teams providing SEI treatment consisting of psychiatrists, psychologists, nurses, social workers, physiotherapists, and vocational therapists. All team members, except for the psychiatrist, function as case managers. | Levels of negative symptoms did not differ between the intervention group and control group. | Participants receiving five years of OPUS treatment were more likely to remain in contact with specialised mental health services, had higher client satisfaction, and had a stronger working alliance than the control group. | Our results differ from previous long term follow-up studies of SEI treatment where participants seem to relapse after end of the intervention treatment. |
| Chen et al. (2015) | Three-year community case management for early psychosis: a randomised controlled study. | Randomized controlled trial; outcomes over 1 year. | Patients who received Early Assessment Service  for Young People with Early Psychosis (EASY) for 2 years were randomized to receive further case management vs. standard care. | n = 160 Cantonese speaking Chinese patients aged 18 to 35 years with a diagnosis of schizophrenia, non-affective psychosis, affective disorders with psychotic features, or delusional disorder who had received EASY for 2 years in Hong Kong. | Community case management involving psychiatric care. | By the end of study, the case management group had better functioning, fewer negative symptoms, general psychopathology, and depressive symptoms, and a trend of fewer defaults from psychiatric consultations. | Social and Occupational Functioning Assessment Scale (SOFAS) and Role Functioning Scale (RFS) were also used. The SOFAS and RFS scores in the case management group improved significantly from baseline to 6 months, and from 6 to 12 months (Table 3). Patients in the case management group attained significant functional improvement, whereas they did not in control group. | Patients were better engaged by having a case manager, probably owing to the extra rapport. |
| Lloyd-Evans et al. (2015) | Evaluation of a community awareness programme to reduce delays in referrals to early intervention services and enhance early detection of psychosis. | Quasi experimental- pre and post intervention questionnaires and mixed methods including qualitative analysis; outcomes at one year post intervention. | A one-year community awareness program about psychosis targeting staff in non-health service community organizations. The program comprised psycho-educational workshops and Early Intervention Services (EIS) link workers, and offering direct referral routes to EIS. | n = 367 staff over 41 workshops from non-health service community-based organizations in London, UK. | Awareness program delivered within the worplace of non-health community-based organizations involved with young people including youth and faith groups, employment, education and housing organisations, black and minority ethnic community groups, probation and social services, and the police in London, UK. | Participants’ knowledge and attitudes to psychosis and attitudes to mental health services improved significantly following workshops. | The community awareness program did not reduce treatment delays for people experiencing first episode psychosis. Further research is needed regarding effective means to reduce duration of untreated psychosis. | None. |
| McFarlane et al. (2015) | Clinical and functional outcomes after 2 years in the early detection and intervention for the prevention of psychosis multisite effectiveness trial. | Quasi experiment (risk based allocation study); outcomes at 6, 12 and 24 months . | Family-aided Assertive Community Treatment (FACT) for clinically high risk (CHR) subjects or those having an early first episode of psychosis (EFEP) vs. community care for clinically lower risk (CLR) subjects. FACT involves a package of interventions consisting of psychoeducational multifamily group (PMFG) therapy, elements of assertive community treatment, supported education and employment, and psychotropic medication. | n = 337 youth (age 12–25) at risk of psychosis were recruited and assigned to treatment groups based on severity of positive symptoms. | 6 USA mental health agencies provided the treatment setting and conducted community outreach and education programs targeted to teachers, school, and college counselors; nurses and social workers; family and pediatric physicians; and psychiatric practitioners, clinics, and hospitals. | FACT was effective in improving positive, negative, disorganized and general symptoms, Global Assessment of Functioning, and global outcome in youth at risk for, or experiencing very early, psychosis. On the primary variable, positive symptoms, after 2 years FACT, were superior to community care (2 df, p < .0001) for both subgroups: CHR (p = .0034) and EFEP (p < .0001) subgroups. No significant differences in rates of conversion to psychosis. | FACT was superior in the Global Test (p = .0007; p = .024 for CHR and p = .0002 for EFEP, vs CLR) and in improvement in participation in work and school (p = .025). | None. |
| Secher et al. (2015) | Ten-year follow-up of the OPUS specialized early intervention trial for patients with a first episode of psychosis. | Randomized controlled trial; outcomes at 10 years post intervention. | Treatment as usual (TAU) (indefinitely) vs. 2 years of OPUS treatment followed by TAU (indefinitely). | n = 547 patients with a first episode of psychosis in Denmark. | Social workers, psychologists, psychiatric nurses, occupational therapists, and a psychiatrist as well as community mental health centers. | Of the 547 participants included in the study, 347 (63.4%) took part in this follow-up. While there was evidence of a differential 10-year course in the development of negative symptoms, psychiatric bed days, and possibly psychotic symptoms in favor of OPUS treatment, differences were driven by effects at earlier follow-ups and had diminished over time. | Statistically significant differences in the course of use of supported housing were present even after 8-10 years. There were no differences between OPUS and TAU regarding income, work-related outcomes, or marital status. | To further investigate the positive effects of OPUS, researchers suggest OPUS treatment should be extended to a period of 5 years. |

| **Community Interventions: School-based Mental Health** | | | | |  |  |  |  |
| --- | --- | --- | --- | --- | --- | --- | --- | --- |
| **Author** | **Title** | **Design** | **Interventions** | **Participants** | **Community Setting/Involvement** | **Mental Health Outcomes** | **Individual or Community-level Social Outcomes** | **Other Outcomes** |
| Antonson et al. (2018) | Upper secondary school students' compliance with two Internet-based self-help programmes: a randomised controlled trial. | Randomized controlled trial; outcome 8 weeks, 3 and 8 months after intervention. | Waiting list vs. one of the two programmes (mindfulness based (iMBI) or music based), on their own incentive during schooltime. | n = 283 upper secondary school students in two schools in Sweden. | Two Swedish upper secondary schools with coordinators at each institution. | There were no associations between the psychiatric and stress-related symptoms at baseline and compliance in any of the intervention groups, and no evidence for differences in compliance in relation to the type of program. | Less than 20 logged into each intervention and only 1 performed a full intervention (iMBI). No significant differences in any of the scales were found between those who logged in and those who did not. The potential effect of Internet-based self-help programmes was not possible to examine due to low compliance rates. | Adolescents seem to have a very low compliance with Internet-based self-help programmes if left to their own incentive. Additional studies are needed to examine how compliance rates can be increased in Internet-based self-help mindfulness programmes in adolescents, as the potentially positive effects of mindfulness are partly related to compliance rates. |
| Bao et al. (2015) | The beneficial effect of Tai Chi on self-concept in adolescents. | Longitudinal study; outcome after 1 year of starting intervention. | Tai Chi sessions vs. China's 8th edition broadcasting gymnastics for 60-minute sessions, five times a week for one year. | n = 160 students from a middle school in China. | Chinese middle school; classes were taught by a martial arts coach of the Tai Chi from Shandong Sports Team. | Statistical analysis shows the significant reduction of anxiety in the experimental group compared with the control group. The results suggest that the Tai Chi intervention could improve self-concept in adolescents. | Statistical analysis shows the significant reduction of good behaviour, intellectual and school status, and popularity in the experimental group compared with the control group. | None. |
| Brotman et al. (2016) | Effects of ParentCorps in Prekindergarten on Child Mental Health and Academic Performance: Follow-up of a Randomized Clinical Trial Through 8 Years of Age. | Cluster randomized clinical trial; outcomes at 3 years post intervention. | ParentCorps: professional development for teachers, MH professionals, paraprofessionals; programs for parents and pre-K students led by mental health professionals and teachers vs. treatment as usual (TAU). | n = 1,050 children, average 4 years old (at enrollment -> 8 years old at follow up), ~50%male, ~70% low-income, ~86% non-Latino Black, with an English speaking caregiver in New York City. | Elementary schools in NYC, serving predominant low-income, black student population. | Relative to their peers in prekindergarten programs, children in ParentCorps-enhanced prekindergarten programs had lower levels of mental health problems. | Intervention in prekindergarten led to better academic performance 3 years later. Teacher-rated academic performance decreased in BOTH conditions, but less for the intervention group. Reading test scores higher for intervention group, no difference in math achievement with no significant interaction of intervention and time. | None. |
| Burckhardt et al. (2015) | A Web-Based Adolescent Positive Psychology Program in Schools: Randomized Controlled Trial. | Randomized controlled trial; Feasibility study. Outcomes at 6 weeks post intervention. | Positive psychology condition, "Bite Back" developed to improve the well-being and happiness of young people, involving positive psychology exercises and information vs. control condition of series of non-psychology entertainment websites. Both interventions delivered online for 6 hours over 4-6 weeks during class time. | Students, aged 12-18 years old, across four Australian high schools (two Anglican girls’ schools, a Catholic boys’ school and a Jewish co-educational school). | Within Australian high schools ranked as high in respect of socioeconomic status. | No significant group differences. Both conditions demonstrated reductions in depression, stress, total symptom scores and improvements in life satisfaction scores post intervention. Only the control condition demonstrated significant increases in flourishing scores post intervention. | None. | None. |
| Burckhardt et al. (2016) | **A randomized controlled trial of strong minds: A school-based mental health program combining acceptance and commitment therapy and positive psychology.** | Randomized controlled trial; outcomes over 3 months. | School-based mental health program combining positive psychology with acceptance and commitment therapy (Strong Minds) vs. Pastoral care (treatment as usual). | n = 267 high school students in year 10 and 11. | 1 Episcopalian high school in New South Wales, Australia (Sydney). | Results demonstrated that compared to controls, participants in the Strong Minds condition with elevated symptom scores (n = 63) reported significant reductions in depression, stress, and composite depression/anxiety symptoms with medium to strong effect sizes. Increased wellbeing in the total sample and decreased anxiety scores for students with elevated symptoms were significant for Year 10 students with medium effect sizes. | Increased wellbeing in the total sample were significant for year 10 students with medium effect sizes. | None. |
| Byford et al (2015) | Cost-effectiveness analysis of a communication-focused therapy for pre-school children with autism: results from a randomised controlled trial. | Randomized controlled trial; outcomes at 13 months. | A parent-mediated, communication-focused therapy (PACT) for children with autism vs. treatment as usual (TAU). | n = 152 pre-school children with autism enrolled in specialist mental health services. | Within mental health outpatient specialist centres in London, Manchester and Newcastle, UK. | Reduction in primary outcome of severity of autism symptoms at 13-month follow-up (measured by the ADOS-G scale) by 3.9 points (SD 4.7) on the ADOS-G in the PACT + TAU group and by 2.9 points (SD 3.9) in the TAU group, representing a between-group effect size of −0.24 (95 % CI −0.59 to 0.11). A clinically meaningful improvement was in evidence for 53 % of the PACT + TAU group compared to 41 % for TAU (odds ratio 1.91, 95 % CI 0.94 to 3.87, p = 0.074). | None. | Economic evaluation of cost effectiveness concluded that PACT is associated with significantly greater costs and no significant difference in outcome, and was not recommended as a cost-effective addition to TAU. |
| Chandler et al. (2015) | Resilience Intervention for Young Adults With Adverse Childhood Experiences. | Experiment; Feasibility study. Outcomes at 4 weeks post intervention and qualitative analysis of participant experience. | A 4-week trauma informed psycho-educational intervention combining resilience training and positive youth development, the 'Empower Resilience Intervention' vs. no intervention. | n = 28 female USA students enrolled in a university undergraduate program. | In university campus location in the US. | No group differences in mental health outcomes as measured by the Resilience Scale (Wagnild, 2009). In the qualitative analysis young adults in the intervention group self reported building strengths, reframing resilience, and creating support connections. | None. | No significant change in health risk behaviors as measured by the Health Behavior Questionnaire. Increase in physical activity observed in the intervention group. |
| Chisholm et al. (2016) | Impact of contact on adolescents' mental health literacy and stigma: the SchoolSpace cluster randomised controlled trial. | Cluster randomized controlled trial; outcomes at 2 week post intervention follow up. | A 1-day school based mental health literacy and stigma educational programme delivered by mental health professionals vs. the educational program plus 'contact' (an additional interactive session with a young person with lived experience of mental illness). | n = 769 secondary school children between the ages of 11 and 13 in the UK. | Within secondary schools in Birmingham, UK. Young lay people with lived experience of mental illness were involved in delivering the 'contact' intervention. | Primary outcome of attitudinal stigma improved in both conditions. Contact with a young person with lived experience reduced the impact of the intervention for a number of outcomes. Significant improvements were found in the education-alone condition compared with the contact and education condition for secondary outcomes of knowledge-based stigma and mental health literacy. | None. | None. |
| Clarke et al. (2015) | Parent attendance and homework adherence predict response to a family-school intervention for children with ADHD. | Randomized clinical trial; outcomes over 12 weeks. | 12-session evidence-based family-school intervention for children with attention-deficit/hyperactivity disorder vs. control group. | n = 92; M age = 9.4 years, SD = 1.27; 67% male, 69% white participants in the United States. | Parent/family and clinican records. | None. | Early homework adherence appeared to be more predictive of outcomes than later adherence, whereas attendance did not predict outcomes during either half of treatment. These results indicate that, even in the context of evidence-based practice, it is the extent to which parents actively engage with treatment, rather than the number of sessions they attend, that is most important in predicting intervention response. | None. |
| Clarke et al. (2017) | School based cognitive behavioural therapy targeting anxiety in children with autistic spectrum disorder: a quasi-experimental randomised controlled trial incorporating a mixed methods approach. | Quasi-experimental randomized controlled trial; outcomes at 6-8 weeks post intervention. | 6 week program with children attending the program once a week for an hour vs. waiting control (receive intervention after all the data has been collected). | n = 28 students with autistic spectrum disorder from schools in England. | School-based cognitive behavorial therapy (CBT) programs incorporating both the parent and the child. | Children in the experimental condition had lower levels of anxiety, maintained at follow-up and changes found in coping behaviours such as lower behavioural avoidance strategies and increased problem solving strategies at follow-up. | None. | None. |
| Coelho et al. (2017) | Promoting a Positive Middle School Transition: A Randomized-Controlled Treatment Study Examining Self-Concept and Self-Esteem. | Randomized-controlled treatment; outcomes over 2 years. | School adjustment program for middle school transition and potential gender differences vs. control group. | n = 1,147 students (M age = 9.62; SD = 0.30, 45.7 % girls) in Lisbon, Portugal. | School-based program. | Following the transition to middle school, students reported lower levels of self-concept (academic, emotional and physical) and self-esteem, while participation in the intervention led to increases in self-esteem and gains in social self-concept. No gender differences were found. | None. | None. |
| Connell et al. (2016) | Long-Term Effects of the Family Check-Up in Early Adolescence on Risk of Suicide in Early Adulthood. | Experiment; outcomes over 6 years. | Family Check-Up, (a school-based prevention program designed to reduce adolescent substance use and behavior problems through improving parenting skills and family functioning and which follows an adaptive intervention framework in which intervention targets and doses are tailored to the individual needs of families) offered as part of a multilevel intervention including: (a) a universal classroom-based intervention, (b) the Family Check-Up and (c) family management treatment (indicated) vs. control. | n = 998 sixth grade adolescents and their families in the northwestern USA. | Delivered in three middle schools within a metropolitan community. | Receipt of the FCU was associated with reductions in suicide risk across adolescence and early adulthood. | None. | None. |
| Cook et al. (2015) | An integrated approach to universal prevention: Independent and combined effects of PBIS and SEL on youths' mental health. | Quasi-randomized control design; outcomes at 5 months. | Two evidence based school-based universal mental health prevention programs, Positive Behavioral Interventions and Supports (PBIS) and Social Emotional Learning (SEL) on student mental health outcomes. Business-as-usual (BAU) vs. PBIS alone vs. SEL alone vs. COMBO condition for 5 months. | n = 191 students from eight 4th and 5th grade classrooms (four from each school) in the Southeastern region of the United States. | Two large elementary schools located in the Southeastern Region of the United States who serve a high proportion of economically disadvantaged youth. | As predicted, the COMBO condition produced significantly greater improvements in overall mental health and reductions in externalizing behaviors when compared to all other conditions. The results also indicated that the PBIS- and SEL-only conditions were both able to produce significant improvements in overall mental health functioning as compared with the BAU control. | None. | Treatment acceptability and feasibility was found across all conditions. |
| Crooks et al. (2015) | Does an evidence-based healthy relationships program for 9th graders show similar effects for 7th and 8th graders? Results from 57 schools randomized to intervention. | Cluster randomized controlled trial; outcomes at 1 month post intervention. | Social and emotional learning (SEL) program conducted by regular classroom teachers vs. control classrooms. | n =1,012 students in the 8th and 9th grade in a Western Canadian province. | Healthy realtionships program conducted in classrooms by teachers in 57 schools; 8th and 9th grade students. | There were significant group differences on three of four outcomes following intervention, including identification of more successful coping strategies. Overall, students learned relevant information and strategies and were able to apply that knowledge to demonstrate critical thinking, suggesting that adapting an evidence-based approach for use with younger students provided similar benefits. | There were significant group differences on three of four outcomes following intervention, including improved knowledge about violence, and critical thinking around the impact of violence. There was no group difference on general acceptance of violence. These findings build a case for 2 years of consecutive evidence-based healthy relationships programming in grades 8 and 9, consistent with best practice guidelines. | None. |
| Devries et al. (2017) | Does the Good Schools Toolkit Reduce Physical, Sexual and Emotional Violence, and Injuries, in Girls and Boys equally? A Cluster-Randomised Controlled Trial. | Two-arm cluster randomized controlled trial with parallel assignment; outcomes at 18 months. | Schools given the Good School Toolkit (behavioral intervention program consisting of T-shirts, books, booklets, posters, and facilitation guides for activities) vs. schools on the wait-list control for 18 months. | Schoolchildren and staff from 42 Ugandan primary schools. | Teachers and their students in Ugandan primary schools. | None. | The Toolkit was associated with an overall reduction in any form of violence from staff and/or peers in the past week towards both male and female students. Injuries as a result of violence from school staff were also lower in male and female students. Although the Toolkit seems to be effective at reducing violence in both sexes, there is some suggestion that the Toolkit may have stronger effects in boys than girls. The Toolkit is a promising intervention to reduce a wide range of different forms of violence from school staff and between peers in schools, and should be urgently considered for scale-up. Further research is needed to investigate how the intervention could engage more successfully with girls. | None. |
| Dowdall et al. (2017) | **The Benefits of Early Book Sharing (BEBS) for child cognitive and socio-emotional development in South Africa: study protocol for a randomised controlled trial.** | Randomized controlled trial; outcome at 6 months post intervention. | Caregivers trained in supportive book sharing program for children vs. wait-list control group. | Caregivers over the age of 18 who live with a child aged between 23 and 27 months in the outskirts of Cape Town, South Africa. | Caregivers and young children living in an area characterized by high levels of poverty, unemployment and violence. | The Benefits of Early Book Sharing (BEBS) trial aims to evaluate the impact of an early parenting intervention on several key risk factors for the development of violence, including aspects of parenting and child cognition, prosocial behaviour, aggression, and socioemotional functioning. The study is being carried out in a LMIC where violence constitutes a major social and health burden. Since the intervention is brief and, with modest levels of training, readily deliverable in LMIC contexts, a demonstration that it is of benefit to both child cognitive and socioemotional development would be of significance. | None. | None. |
| Dray et al. (2017) | Effectiveness of a pragmatic school-based universal intervention targeting student resilience protective factors in reducing mental health problems in adolescents. | Cluster-randomized controlled trial; outcomes at 3 years post intervention. | Universal, school-based intervention targeting resilience protective factors in reducing mental health problems in adolescents vs. control (provided with no intervention resources or support). | n = 2,149 students aged 12-16 years old in socio-economically disadvantaged areas of NSW, Australia. | 32 secondary schools administering self-reported questionnaires to school children in Australia. | There were no significant differences between groups at follow-up for three mental health outcomes: total Strengths and Difficulties Questionnaire (SDQ), internalizing problems, and prosocial behaviour. A small statistically significant difference in favour of the control group was found for externalizing problems. | Findings highlight the continued difficulties in developing effective, school-based prevention programs for mental health problems in adolescents. | None. |
| Duncombe et al. (2016) | Comparing an Emotion- and a Behavior-Focused Parenting Program as Part of a Multsystemic Intervention for Child Conduct Problems. | Experiment; outcomes at 6 months. | Comparison of an emotion- and a behavior-focused parenting program for children with emerging conduct problems: Tuning in to Kids (TIK) vs. Positive Parenting Program (PPP) vs.  waitlist control. | n = 320 predominantly Caucasian 4- to 9-year-old children from 373 children from 41 schools screened for disruptive behavior problems and deemed at risk for conduct disorder in study setting of Northern Melbourne and Bendigo, Australia. | Both interventions involved group parenting sessions in schools and encouragement to apply strategies in both home and community settings | Both interventions were equally effective in reducing child conduct problems with effects showing statistical and clinical significance. | None. | None. |
| Fishbein et al. (2016) | Short-Term Intervention Effects of the PATHS Curriculum in Young Low-Income Children: Capitalizing on Plasticity. | Experiment; outcomes after 1 academic year. | Schools randomly assigned to (PATHS) vs. attentional control condition. | n = 327 children in kindergarten classrooms with provided caregiver consent in Baltimore City. | Teachers and their students in a city in the US. | Teacher rated behavioral measures (social competence scale, teacher observation of child adaptation revised), student teacher relationship scale, peer relations questionnaire, peer nominations. Findings showed children who received the PATHS curriculum exhibited greater improvements than control students across all teacher-rated behavioral measures of social competence (i.e., emotion regulation, prosocial behavior, peer relations) and behavioral problems (i.e., aggression, internalizing behaviors, impulsivity and hyperactivity) at post-test. | Academic data (4 items from the academic competence evaluation scale). Academic Skill total improved more for the PATHS group. | Children who received PATHS exhibited significantly greater improvements than control students across all teacher-rated behavioral measures such as cognitive functioning, motor impulsivity, delayed gratification, behavioral inhibition, facial emotional recognition. |
| Flock et al. (2015) | Promoting prosocial behavior and self-regulatory skills in preschool children through a mindfulness-based Kindness Curriculum. | Randomized controlled design; outcomes at 12 weeks. | Mindfulness-based “Kindness Curriculum” (KC) intervention vs. a wait-list control group for 12 weeks. | n = 68 preschool children in a medium-sized Midwestern city in the United States. | Preschool program consisting of students and their teachers. | The KC intervention group showed greater improvements in social competence and earned higher report card grades in domains of learning, health, and social-emotional development, whereas the control group exhibited more selfish behavior over time. Interpretation of effect sizes overall indicate small to medium effects favoring the KC group on measures of cognitive flexibility and delay of gratification. Baseline functioning was found to moderate treatment effects with KC children initially lower in social competence and executive functioning demonstrating larger gains in social competence relative to the control group.These findings, observed over a relatively short intervention period, support the promise of this program for promoting self-regulation and prosocial behavior in young children.They also support the need for future investigation of program implementation across diverse settings. | None. | None. |
| Gigantesco et al. (2015) | A Universal Mental Health Promotion Programme for Young People in Italy. | Experiment; outcomes at 2 months. | A school based universal mental health promotion program based on a structured handbook delivered one-hour a week for a total of 20 hours of classroom time vs. curricula as usual (control). | n = 308 students average age 15.2 years in 9 Italian high schools completed preintervention test and post intervention test. | Teaching students coping skills which they could practice both at home and in their secondary schools in Italy. | The intervention was associated with a significant improvement in the overall psychological well-being and in life satisfaction scores. | None. | None. |
| Gold et al. (2017) | Group Music Therapy as a Preventive Intervention for Young People at Risk:Cluster-Randomized Trial. | Cluster-randomized trial; outcome at 3 months. | Weekly sessions over 8 weeks of group music therapy vs. self-directed music listening. | n = 100 students with self-reported unhealthy music use in Victoria, Australia. | Music coordinators and their students in government-funded secondary schools in socio-economically disadvantaged areas. | Both interventions were well accepted. No effects were found between group music therapy (GMT) and self-directed music listening (SDML) (all p > 0.05); both groups tended to show small improvements over time. Younger participants benefited more from GMT, and older ones more from SDML (p = 0.018). | None. | None. |
| Goossens et al. (2016) | Effectiveness of a brief school-based intervention on depression, anxiety, hyperactivity, and delinquency: a cluster randomized controlled trial. | Cluster randomized controlled trial; outcomes at 2, 6, and 12 months post intervention. | Preventure, a school-based, selective preventive intervention, tailored to four personality types vs. the no intervention control group for 90 minutes over two weeks. | n = 699 high risk students (mean age 14 years). | 15 High Schools in Netherlands conducting the intervention program. | No significant intervention effects were found on 22 of the 24 tests. A positive intervention effect on anxiety was found in the anxiety sensitivity personality group at 12-month follow-up, and a negative intervention effect on depression was found at 12-month follow-up in the negative thinking group. In post hoc growth curve analyses these effects were not found. This study found no convincing evidence for the effectiveness of Preventure in The Netherlands on mental health problems. This finding is not in line with the results of an earlier effectiveness study in the UK. This highlights the need for more research into the knowledge transfer model of interventions, to ensure that interventions are effective in a variety of circumstances. | None. | None. |
| Graziano et al. (2016) | Beyond behavior modification: Benefits of social-emotional/self-regulation training for preschoolers with behavior problems. | Experiment; outcomes at 6 months post intervention | First intervention package was an 8-week School Readiness Parenting Program (SRPP). Families randomized into the second and third intervention packages received not only the weekly SRPP, but children also attended two different versions of an intensive kindergarten summer readiness class (M-F, 8a.m.-5p.m.) that was part of an 8-week summer treatment program for pre-kindergarteners (STP-PreK). One version included the standard behavioral modification system and academic curriculum (STP-PreK) while the other additionally contained social-emotional and self-regulation training (STP-PreK Enhanced). | The final participating sample consisted of 45 preschool children (76% boys) with at-risk or clinically elevated levels of EBP (externalizing behavior problems) in large urban southeastern city in the U.S. with a large Hispanic/Latino population. | Kindergarten students, their families, and the school based program. | Children in the STP-preK Enhanced group had greater growth across time in emotion knowledge, emotion regulation, and executive functioning. Children's behavioral functioning significantly improved across all 3 groups. | Children in the STO-Prek Enhanced group experienced significantly steeper growh in academic acheivement across time compared to children in the other two groups. | None. |
| Gumz et al. (2017) | Efficacy of a prevention program for eating disorders in schools: a cluster-randomized controlled trial. | Longitudinal cluster-randomized controlled trial; outcomes 6 months post intervention. | Universal prevention program conducted during 6 school hours vs. the no treatment control condition. | n = 2,342 students at all levels of eating disorder risk in 23 schools from 8th or 11th grade in Germany. | Program conducted in secondary schools in Germany. | We do believe that knowledge of mental illnesses should be an integral part of education and that prevention programs might also contribute to destigmatization and early detection of mental diseases. | In summary, the present prevention program can be recommended to older adolescents where it resulted in increased knowledge on eating disorders and a decrease in eating disorder pathology. We do believe that knowledge of mental illnesses should be an integral part of education and that prevention programs might also contribute to destigmatization and early detection of mental diseases. | None. |
| Guo et al. (2015) | A Longitudinal Evaluation of the Positive Action Program in a Low-Income, Racially Diverse, Rural County: Effects on Self-Esteem, School Hassles, Aggression, and Internalizing Symptoms. | Longitudinal study; outcomes at 1, 2, 3, and 4 years post intervention. | Positive action, a school-based program, that aims to decrease problem behaviors (e.g., violence, substance use) and increase positive behaviors (e.g., school engagement, academic achievement) vs. no program for 3 years. | n = 1,246 middle school youth (52% female) of ethnically/racially diverse backgrounds (27% White, 23% African American, 12% mixed race/other, 8% Latino, 30% as American Indian) (age range 9-20) located in two violent, low-income rural counties in North Carolina. | Program conducted in rural middle school. | The results indicate that the program generates statistically significant beneficial effects for youth from the intervention county on self-esteem scores and school hassles scores. Although the program generates beneficial effects for intervention youth on the change in aggression scores, the finding is not statistically significant. The finding on the change in internalizing scores shows a non-significant detrimental effect: the youth from the comparison county have lower internalizing scores than those from the intervention county. | The results indicate that the program generates statistically significant beneficial effects for youth from the intervention county on self-esteem scores and school hassles scores. | None. |
| Guo et al. (2017) | Does Depression Screening in Schools Reduce Adolescent Racial/Ethnic Disparities in Accessing Treatment? | Cluster randomized design; outcomes after one academic school year. | Schools administer universal depression screening in the seventh and eighth grades using the Patient Health Questionnaire for Adolescents (PHQ-A) vs. schools assigned to the control condition with no mental health screening but referrals to care as usual. | n = 2,494 Asian American and Latino students in the 7th and 8th grade in medium-sized urban school district in Southern California | Schools that either conducted or did not conduct universal depression screening. | None. | Multilevel analyses showed that enrollment in a universal screening school, Latino ethnicity, and low academic performance were associated with greater likelihood of referral. However, these factors were not related to caregiver consent or treatment initiation. Screening-triggered referrals were less likely to result in caregiver consent compared to routine referrals. Furthermore, universal screening did not result in a statistically significant reduction in racial/ethnic disparities in treatment referral. | None. |
| Havighurst et al. (2015) | An emotion-focused early intervention for children with emerging conduct problems. | Randomized control trial; outcomes 10 months post intervention. | Emotion socialization parent program (TIK) plus a child program and a school intervention vs. waitlist control (delayed start of intervention by 1 year). | n = 204 primary caregivers and their children (Mage = 7.05, SD = 1.06; 74 % boys). Children in the first 4 years of elementary school in Australia. | Schools in lower socioeconomic areas of Victoria, Australia. | Results showed intervention parents but not controls became less emotionally dismissive and increased in empathy, and children showed better emotion understanding and behavior compared to control children. These outcomes lend support for an emotion-focused approach to early intervention in a real-world context for children with conduct problems. | These outcomes lend support for an emotion-focused approach to early intervention in a real-world context for children with conduct problems. | None. |
| Ho et al. (2017) | A Sports-Based Youth Development Program, Teen Mental Health, and Physical Fitness: An RCT. | Randomized controlled trial; outcomes at 1 month post intervention. | Positive Youth Development (PYD)-based sports mentorship program vs. control (exclusive access to health education website). | n = 646 students from 12 secondary schools, mean age 12 years old, 58% girls, Chinese-speaking, engaged in 2 extra-curricular activities in Hong Kong. | 12 secondary schools in 4 districts. | Intervention group had greater mental well-being, self-efficacy, and resilience. | Intervention group had improved lower limb muscle strength, flexibility, balance, and physical activity levels. | None. |
| Hutchings et al. (2016) | Evaluating the Incredible Years Toddler Parenting Programme with parents of toddlers in disadvantaged (Flying Start) areas of Wales. | Randomized controlled trial; outcomes at 6 months and 12 months after starting intervention. | IYTPP 12-session program vs. wait-list control (offered intervention after the 6 month follow-up). | n = 89 parent-child dyads; child aged between 12 and 36 months and their families from 8 FS sites across North, Mid, and South Wales. | Flying start (new project in Wales for pre-school children and parents living in highly targeted disadvantaged areas identified by the Welsh government. | None. | Quality of home environment and child development was significantly improved at 12 months compared to baseline and compared to 6 months. | None. |
| Johnson et al. (2017) | A randomized controlled evaluation of a secondary school mindfulness program for early adolescents: Do we have the recipe right yet? | Randomized controlled trial; outcomes at 6 and 12 months. | Nine lesson curriculum delivered by an external facilitator vs. without parental involvement vs. allocated to a usual curriculum control group. | Students (Mage 13.44, SD 0.33; 45.4% female) with (N = 191) or without (N = 186) parental involvement, or were allocated to a usual curriculum control group (N = 178) in Australia | Four urban coeducational secondary schools. | There were no differences in outcomes between any of the three groups at post-intervention, six or twelve month follow-up. A wide range of moderators were examined but none impacted outcomes. | None. | None. |
| Kiviruusu et al. (2016) | Short-term effects of the "Together at School" intervention program on children's socio-emotional skills: a cluster randomized controlled trial. | Cluster randomized controlled trial; outcomes over 2 years. | Together at School intervention--a universal intervention program that promotes SEL skills among primary age children, using a whole school approach and delivered by teachers vs. control group receiving two 3-hour lessons by the research group over two school years. | n = 3,704 children; 79 primary schools (40 intervention and 39 control) in Finland. | Teachers delivering intervention program to their students at 79 primary schools (40 intervention). | Short-term results of the intervention program did not show any main effects on children's socio-emotional skills or psychological problems. | None. | None. |
| Langer et al. (2017) | **[Effects of a mindfulness intervention in Chilean high schoolers].** | Experimental, randomized controlled trial; outcomes at 3 and 6 months post intervention. | 8 weekly 44-minute mindfulness intervention sessions vs. waiting list control. | n = 88 teenagers aged 13 ± 0.6 years (46 females) in Chile. | Two schools in Chile; students received notebook with mindfulness activities. | These preliminary results suggest the feasibility and effectiveness of a mindfulness intervention in Chilean schools as a strategy to reduce negative emotional states and prevent risk factors in adolescent population groups. | None. | None. |
| Langley et al. (2015) | Bounce back: Effectiveness of an elementary school-based intervention for multicultural children exposed to traumatic events. | Experiment. Feasibility and acceptability analysis; outcomes at 3 and 6 months. | Bounce Back, a 10-session cognitive-behavioral group intervention delivered by school clinicians vs. delayed (3-month waitlist) Intervention. Group and individual sessions used to deliver the intervention alongside parent education sessions. | n = 74 ethnically diverse school children (grades 1-5, mean age 7.65) with moderate or higher levels of PTSD symptoms (>20 on PTSD Reaction Index) and experience of one or more traumatic events and their primary caregivers. | Delivered within 12 schools in Los Angeles County USA, by school based clinicians. | Improvements in primary outcomes of parent- and child-reported post traumatic stress and anxiety symptoms maintained or showed continued gains over the 3-month follow-up period. Improvements in these domains and depression were observed in the delayed intervention group. Some positive influences on secondary functional outcomes (social adjustment, behavior, emotion expression, and coping) were observed. | None. | None. |
| Lee et al. (2016) | School-Based Interventions for Anxious Children: Long-Term Follow-Up. | Randomized clinical trial; outcomes at 3, 6, 12 months, 2, and 3 years posttreatment. | Group cognitive-behavioral therapy (CBT) for children vs. group CBT for children plus parent training vs. no-treatment control for 9 weeks. | n = 61 nonclinical sample of anxious children and their parents in the United States. | School-based intevention involving both schoolchildren and their parents. | When the two CBT groups were combined and compared with control, the combined treatment group showed significantly greater reduction in children's anxiety severity based on the parent ratings in the first longitudinal phase. However, on the parent Clinician Severity Rating, gains were maintained to 3 years. Child report revealed no significant differences between groups on anxiety reduction. From parental perspective only, school-based group CBT appeared to be beneficial in decreasing severity of anxiety symptoms and maintaining gains over time. | None. | None. |
| Lochman et al. (2015) | Counselor-Level Predictors of Sustained Use of an Indicated Preventive Intervention for Aggressive Children. | Longitudinal study; outcomes after 2 years. | School counselors who were trained to implement the Coping Power program (CP) with at-risk aggressive students in the context of a dissemination field trial. | n = 38 school counselors (97% female and 53% African American). | School counselors completed surveys and teachers provided data on student behavior pre- and post-intervention. | None. | The results indicated that counselors' perceptions of interpersonal support from teachers within their schools, their perceptions of the effectiveness of the program, and their expectations for using the program were all predictive of program use over the following 2 years. In addition, certain counselor personality characteristics (i.e., conscientiousness) and the level of actual teacher-rated behavior change experienced by the children they worked with during training were predictors of counselors' use of the program during the second year after training. These results indicate the central importance of teacher support and of child progress during training in the prediction of counselors' sustained use of a prevention program. | None. |
| Lonigan et al. (2015) | Impacts of a Comprehensive School Readiness Curriculum for Preschool Children at Risk for Educational Difficulties. | Cluster-randomized study; outcomes at 8 months. | Business-as-usual condition vs. explicit socioemotional curriculum vs. implicit socioemotional curriculum for one school year. | n = 110 classroom teachers from randomized classrooms and approximately eight students from each classroom (n = 760) who averaged 4.48 (SD = 0.44) years of age at the start of the school year in Florida and Texas. | Pre-school teachers and their students. | There were positive impacts of the two versions of the curriculum on language, phonological awareness, math, and socioemotional outcomes. | There were no added benefits to academic or socioemotional outcomes for the children receiving explicit socioemotional instruction. | None. |
| Low et al. (2015) | Promoting social-emotional competence: An evaluation of the elementary version of Second Step®. | Randomized controlled trial; outcome 6 months after intervention. | 4th Edition of Second Step (SEL program) combined with a brief training on proactive classroom management vs. business-as-usual classroom management. | n = 321 teachers, 7,300 students in Kindergarten to 2nd grade in 61 schools across six school districts in Arizona and Washington state. | Program delivered in school and teachers provided data regarding their students. | The majority of significant findings were moderated effects, with 8 out of 11 outcome variables showing significant improvements in social-emotional competence and behavior for children who started the school year with skill deficits relative to their peers in the interventiong group. Data was based on teacher report. | None. | None. |
| Luxford et al. (2017) | Evaluating the Effectiveness of a School-Based Cognitive Behavioural Therapy Intervention for Anxiety in Adolescents Diagnosed with Autism Spectrum Disorder. | Randomized control trial; outcomes at 6 weeks post intervention. | 6 sessions of the Exploring Feelings Cognitive Behavioural Therapy (CBT) intervention vs. wait-list control group. | n = 35 pupils from four mainstream secondary schools located in the south-east of England. Participants were required to have a formal diagnosis of ASD from a qualified health professional (N = 26 adolescents had a formal diagnosis of ASD and N = 9 had a diagnosis of Asperger’s Syndrome). | Mainstream secondary schools in England. | For intervention group, greater reductions in anxiety symptoms, school anxiety and social worry, as reported by parents, teachers and young people themselves, and these results were maintained at a 6 week follow-up. | Teachers reported marginally increased social responsiveness for young people in the intervention group, was most evident 6 weeks post-intervention. | None. |
| Mackay et al. (2017) | A Pilot Randomised Controlled Trial of a School-Based Resilience Intervention to Prevent Depressive Symptoms for Young Adolescents with Autism Spectrum Disorder: A Mixed Methods Analysis. | Mixed methods pilot randomized control trial; outcomes at 6 months post intervention. | RAP-A-ASD (Resourceful Adolescent Program-Autism Spectrum Disorder) - adapted from RAP-A program (for ASD population) - 11, 50min weekly sessions coving variety of topics related to self regulation and interpersonal function; delivered in school by trained facilitator (provisional psychologist / intern) vs. treatment as usual control group receiving support from school-based services. | N = 29, ~11 year old, Australian children (~90% males) with high functioning ASD (from 18 schools), ~50% with depression at baseline in Brisbane, Australia. | 18 schools providing treatment services. | Quantitative results showed significant intervention effects on parent reports of adolescent coping self-efficacy (maintained at 6 month follow-up) but no effect on depressive symptoms or mental health. Qualitative outcomes reflected perceived improvements from the intervention for adolescents’ coping self-efficacy, self-confidence, social skills, and affect regulation. | None. | None. |
| McNaughton et al. (2015) | Efficacy of a Latino mother-child communication intervention in elementary schools. | Experiment; outcomes over 24 weeks. | An adapted mother–child communication intervention program comprising 6 x 2 hour sessions of manual-guided, mental health promotion, communication, skill-building sessions with mother-child dyads vs. no intervention. | n = 53 mother–child dyads of Mexican immigrant mothers and their fourth to sixth grade children in a large Midwestern city in the USA. | Delivered after school in four urban elementary schools located in ethnically diverse, low income neighbourhoods in a large Midwestern city in the USA. Data collected at weekends in community locations convenient to families. | Intervention children reported Improvements in child depressive symptoms and health self-concept with small/moderate effect sizes (d =.29 and .36, respectively) as compared to control children. | None. | Improvements in children’s reports of problem-solving communication, with their mother and mothers’ reports of reduced family conflict in intervention group. Effect size differences were small to moderate. |
| Melnyk et al. (2015) | Twelve-Month Effects of the COPE Healthy Lifestyles TEEN Program on Overweight and Depressive Symptoms in High School Adolescents. | Experiment; outcomes at 12 months. | Healthy lifestyle interventions for teens: The COPE (Creating Opportunities for Personal Empowerment) Healthy Lifestyles TEEN (Thinking, Emotions, Exercise, Nutrition) program vs. an attention control program (Healthy Teens). The COPE/Healthy Lifestyles TEEN program is a manualized 15 week, educational and cognitive-behavioral skills building program with physical activity integrated into a health course and taught by teachers. | n = 779 14-16 year old adolescents in the US Southwest. | Within eleven high schools in 2 school districts in the Southwest Region of the United States. | Reduction in depression symptoms for youth who began the study with extremely elevated depressive symptoms (measured on depression subscale of the Beck Youth Inventory II© (BYI-II). The intervention group COPE teens had significantly lower depression at 12 months compared to Healthy Teens (COPE M=42.39; Healthy Teens M=57.90); (F1, 12 = 5.78, p = .03). | None. | The intervention COPE teens had a significantly lower BMI at 12 months (F1, 698 = 11.22, p = .001) than Healthy Teens (24.95 versus 25.48). There was a significant decrease in the proportion of overweight and obese COPE teens from baseline to 12 months (χ2= 5.40, p = .02) as compared to Healthy Teens. |
| Milin et al. (2016) | Impact of a Mental Health Curriculum on Knowledge and Stigma Among High School Students: A Randomized Controlled Trial. | Randomized controlled trial; outcome at 2 weeks post intervention. | Curriculum incorporated into the grade 11 and 12 "Healthy Living" courses and teacher delivered vs. control condition (teaching as usual). | n = 534 students from 24 high schools and in the regional area of Ottawa, Ontario, Canada. | Teachers delivering intervention curriculum in high schools. | Signifciant change in stigma scores over time, and positive attitudes increasing from pre to post. Significant change in knowledge scores over time as well. Increases in knowledge significantly predicted increases in positive attitudes towards mental health. | None. | None. |
| Miller et al. (2014) | A school health center intervention for abusive adolescent relationships: a cluster RCT. | Cluster randomized controlled trial; outcomes at 3 months. | Counselors at school health centers were trained in adolescent relationship abuse (ARA) vs. standard care control group. | n = 1,062 eligible students ages 14 to 19 years in Northern California. | 8 school health centers (SHCs) involving counselors and students. | Intervention versus control adjusted mean differences on changes in primary outcomes were not statistically significant: recognition of abuse, intentions to intervene and knowledge of resources. Intervention participants had improved recognition of sexual coercion compared with controls. In exploratory analyses adjusting for intensity of intervention uptake, intervention effects were significant for increased knowledge of relationship abuse resources and self-efficacy to use harm reduction behaviors. Among participants reporting relationship abuse at baseline, intervention participants were less likely to report such abuse at follow-up. Adolescents in intervention clinics who reported ever being in an unhealthy relationship were more likely to report disclosing this during the SHC visit. This is the first evidence of the potential benefit of a SHC intervention to address abusive relationships among adolescents. | None. | None. |
| Molnar et al. (2017) | [Behavioral intervention for preschool children with autism – outcome of parent-based Intervention]. | Waiting-list control experiment; outcome after 1 year. | Children received 1 year of home-based early intensive behavioral intervention (EIBI) for approximately 20 hours a week, their parents functioning as primary therapists vs. the waiting-list control group. | n = 13 children with autism spectrum disorder (ASD) between 2 and 5 years of age at intake participated in the study. | Home setting; parents of children with ASD and children with ASD. | After 1 year of EIBI, we found a significant increase in the PEP-3 scores and MDI scores as well as a significant reduction in the CARS 2 scores. No significant changes were seen when participants were on the waiting list. The stress level of the parents did not change significantly and in fact showed overall a slight decrease. | None. | None. |
| Nix et al. (2016) | The randomized controlled trial of Head Start REDI: Sustained effects on developmental trajectories of social-emotional functioning. | Randomized controlled trial; outcomes at end of intervention (1 year) and then annually for a further 4 years. | The 'Head Start REDI' intervention (Research-based, Developmentally-Informed). A one year enriched preschool curriculum involving integrated language, emergent literacy and social-emotional skills and enhanced support for positive teaching practices vs. 'Head Start' as usual. | n = 356 children recruited in final year of pre-school who attended Head Start in three Pennsylvania counties. 70% of the children came from families living in poverty. | Intervention delivered by teachers within primary school setting in the USA. | Impact of sustained effects on children’s developmental trajectories of social-emotional functioning four years after participation were measured using multiple scales. Children in the intervention group were statistically significantly more likely to exhibit the most optimal developmental trajectories of social competence, aggressive-oppositional behavior, learning engagement, attention problems, student-teacher closeness, and peer rejection. | None. | None. |
| Parisi et al. (2015) | Impact of Experience Corps(®) participation on school climate. | Randomized controlled trial; outcomes at 1 and 2 years. | Experience Corps program (teams of older adult volunteers were placed in high intensity (>15 hours per week), meaningful roles in public elementary schools, to improve the educational outcomes of children as well as the health and well-being of volunteers) vs. usual volunteering opportunity (waitlist control). | n = 25 public schools; adult volunteers and school children in Baltimore City. | 25 Baltimore City public elementary schools. | None. | During the first year of EC participation, school climate was perceived more favorably among staff and students in EC schools as compared to those in comparison schools. However, with a few notable exceptions, perceived school climate did not differ for staff or students in intervention and comparison schools during the second year of exposure to the EC program. These findings suggest that perceptions of school climate may be altered by introducing a new program into elementary schools; however, research examining how perceptions of school climate are impacted over a longer period is warranted. | None. |
| Park et al. (2016) | **Service Use Findings from the Child STEPs Effectiveness Trial: Additional Support for Modular Designs.** | Longitudinal randomized trial; outcomes at 1 and 2 years. | Standard manualized treatment (Standard) vs. modular treatment (Modular) vs. usual care (UC). | n = 174, averaged 10.59 years (SD = 1.76) in age and were predominately male (70%); 45% were Caucasian, 32% were of mixed ethnicity, 9% were African American, 6% were Latino or Hispanic, and 4% were Asian American or Pacific Islander in Honolulu, HI and Boston, MA. | Community mental health settings. | Results showed that youths who received Modular accessed fewer service settings at their one-year follow-up relative to youths who received Standard or UC. Findings suggest that modular treatment may offer an advantage over standardized treatment manuals and UC in terms of sustained clinical benefits, and highlight the importance of treatment design considerations for service systems. | Results showed that youths who received Modular accessed fewer service settings at their one-year follow-up relative to youths who received Standard or UC. | None. |
| Pearce et al. (2016) | Effectiveness of school-based humanistic counselling for psychological distress in young people: Pilot randomized controlled trial with follow-up in an ethnically diverse sample. | Pilot randomized controlled trial; outcomes at 6 and 9 months. | School-Based Humanistic Counseling (SBHC) by School-based couselors with up to 12 sessions of 45 min vs. usual care (UC). | N = 64 young people, M = 14 years old, 78% POC, with elevated levels of emotional distress at baseline (Strengths and Difficutlies Questionnaire) in the UK. | 3 urban secondary schools involving school counselors and school children in the UK. | SBHC resulting in greater reduction in psychological distress and emotional symptoms early in treatment, but without significance over long term follow up. | None. | None. |
| Pelleboer-Gunnink et al. (2015) | Effectiveness and moderators of the preventive intervention kids in divorce situations: A randomized controlled trial. | Randomized controlled trial; outcomes at 6 months and 1 year post intervention. | School-based, child-directed prevention program Kids In Divorce Situations (KIDS) vs. control condition (only receive a letter and factsheet about the study). | n = 156 children; 131 mothers and 76 fathers in the Netherlands. | School and family setting. | Latent growth analyses demonstrated that the intervention significantly reduced child-reported emotional problems and enhanced child-reported communication with the father and mother-reported communication with the child. The effect sizes ranged from .30-.63. Few moderation effects of gender, time since divorce, or perceived parental conflict on the intervention effects were found. After parental divorce, a limited school-based intervention for children can be efficacious in promoting children's emotional well-being and parent-child communication. | None. | None. |
| Piek et al. (2015) | Does the Animal Fun program improve social-emotional and behavioural outcomes in children aged 4-6 years? | Experiment; outcomes at 6 months and 18 months. | A pre-school / kindergarten universal program called Animal Fun (Piek et al., 2010) vs. normal school curriculum. Animal Fun is administered by teachers and through fun design (participants imitating animal movements) aims to improve engagement in physical activity participation and promote fine and gross motor skills and social development in children aged 4–6 years. | n = 511 children (257 boys and 254 girls) in pre-primary clases between ages 4 years 10 months and 6 years 2 months of age in Australia. | Within 12 schools across both metropolitan and regional Western Australia in low socio-economic areas. | Significant improvement in prosocial behaviour of children in the intervention group at 6 and maintained at 18 months. Total difficulties decreased at 6 months for the intervention group, with no change at 18 months. This effect was present only for the hyperactivity/inattention subscale. | None. | None. |
| Puskar et al. (2015) | Testing the 'Teaching Kids to Cope with Anger' Youth Anger Intervention Program in a Rural School-based Sample. | Randomized controlled trial; outcomes at 6 months, and at 1 year post-intervention. | Teaching Kids to Cope with Anger (TJC-A) program, one class period a week 8 weekly sessions vs. control group attending regularly scheduled classes. | n = 179 youths of 14-18 years of age, from three rural high schools. | Rural high schools. | Through analysis of the Anger Index sub-scale of the STAXI-2 at 1 year post-intervention, a significant difference was reported between the control and intervention group. Participants reported that the TKC-A intervention was helpful in coping with emotional, behavioral, and social aspects of anger. Future research may utilize the TKC-A with youth who have anger management problems. Psychiatric-mental health nurses can screen youth for anger and be cognizant of coping skills of youth, assess for anger problems and provide health education to youth about approaches for coping with anger. | None. | None. |
| Roberts et al. (2014) | The Impact of Intensive Reading Intervention on Level of Attention in Middle School Students. | Longitudinal randomized study; outcomes over 3 years. | Reading intervention intensity varied based on student response vs. business as usual for three years. | Middle school students (54% male, 18% limited English proficient, 85% eligible for free or reduced-price lunch, 58% African American, and 32% Hispanic) in two large urban cities in the United States. | Researchers hired teachers to implement intervention program in middle schools. | Findings indicate that intensive, response-based reading intervention over 3 years improved reading achievement and behavioral attention in middle school struggling readers, with treatment directly affecting reading, which in turn influenced attention. In the business-as-usual condition, there was no relation between improved reading and attention. The results are consistent with a correlated liabilities model of comorbidity. The results do not align with the inattention-as-cause hypothesis, which predicts that reading intervention should not affect attention. The findings do not support, but do not necessarily preclude, the phenocopy hypothesis. The results are especially pertinent for older students who may be inattentive partly because of years of struggling with reading. | None. | None. |
| Saarento et al. (2015) | Reducing bullying and victimization: student- and classroom-level mechanisms of change. | Longitudinal study; outcomes after 1 year. | KiVa antibullying program vs. waitlist group (offered an opportunity at their discretion to initiate the KiVa program after 1 year of serving as control schools). | n = 7,491 students (49.5% boys) in Finland. | Nested within 421 classrooms in 77 schools. At the beginning of program implementation, the children were in grades 4, 5, and 6 (mean age 11.3 years). | At the student level, antibullying attitudes and perceptions regarding peers' defending behaviors and teacher attitudes toward bullying mediated the effects of KiVa on self-reported bullying perpetration. The effects on peer-reported bullying were only mediated by antibullying attitudes. At the classroom level, the program effects on both self- and peer-reported bullying were mediated by students' collective perceptions of teacher attitudes toward bullying. Also, perceived reinforcing behaviors predicted bullying but did not emerge as a significant mediator. Finally, bullying mediated the effects of the classroom-level factors on victimization. These findings enhance knowledge of the psychosocial developmental processes contributing to bullying and victimization and shed light on the key mechanisms by which school bullying can successfully be counteracted. | None. | None. |
| Sælid et al. (2017) | Rational emotive behaviour therapy in high schools to educate in mental health and empower youth health. A randomized controlled study of a brief intervention. | Randomized control trial; outcomes at 6 months post intervention. | Rational Emotive Behavior Therapy (REBT) vs. Individual Attention Placebo (ATP) vs. no treatment. | n = 62 young people, 16-19 years old in Norway. | 1 high school in Norway. | REB resulted in reduction in depression and anxiety, dysfunctional thinking, hope, and self esteem for variable durations. | None. | None. |
| Sayal et al. (2016) | Effectiveness and cost-effectiveness of a brief school-based group programme for parents of children at risk of ADHD: a cluster randomised controlled trial. | Cluster-randomized controlled trial; outcomes at 6 months. | School based group program for parents of children at risk of ADHD vs. combined (parent + teacher) intervention vs. no intervention control. | n = 199 primary school children (4-8 years old) at risk of ADHD as determined by high levels of parent-rated hyperactivity/inattention (scoring ≥6 for hyperactivity/inattention on the Strengths and Difficulties Questionnaire (SDQ) (Goodman 1999)). | Interventions were either delivered at the participating 12 mixed sex primary schools or delivered at alternative local venues in the east Midlands, UK. | There was no effect of the parent-only (mean difference = -1.1, 95% CI -5.1,2.9; p = 0.57) or combined interventions (mean difference =-2.1, 95% CI -6.4,2.1; p = 0.31) on the ADHD index. The combined intervention was associated with reduced parent-reported hyperactivity symptoms (mean difference =-5.3; 95% CI -10.5,-0.01; p = 0.05) and the parent-only intervention with improved parental mental health (mean difference =-1.9; 95% CI -3.2, -0.5; p = 0.009). | None. | Cost effectiveness analysis was conducted. Above a willingness-to-pay of £31 per one-point improvement in the ADHD index, the parent-only program had the highest probability of cost-effectiveness. |
| Schell et al. (2015) | Preventing Behavioral Disorders via Supporting Social and Emotional Competence at Preschool Age. | Cluster-randomized controlled trial; outcomes at 5 months post intervention. | Social training program (Lubo from Outer Space, Preschool Version: strengthen emotional knowledge and regulation, the ability to take another person's point of view, communication skills, and social problem solving) vs. business-as-usual control group (conventional kindergarten activities). | n = 221 children at 15 kindergartens, aged 5-6 years in Cologne, Germany. | Kindergartens. | Five months after the end of the intervention, the social-cognitive problem solving strategies of the children in the intervention group had improved more than those of the children in the control group: the intergroup difference in improvement was 0.79 standard deviations of the Wally test (95% confidence interval [CI] 0.13-1.46). This effect was just as marked 5 months later (0.63, 95% CI 0.03-1.23). Prosocial behavior, as measured by the PSBQ, also improved more in the intervention group, with an intergroup difference of 0.37 standard deviations (95% CI 0.05-0.71). | None. | Randomization was unsuccessful: the children in the intervention group performed markedly worse on the tests carried out before the intervention or a control group, in a 2:1 ratio. |
| Schilling et al. (2016) | The SOS Suicide Prevention Program: Further Evidence of Efficacy and Effectiveness. | Randomized control design; outcomes at 3 months post intervention. | The signs of Suicide (SOS) prevention program; a school-based suicide prevention program where students are taught warning signs of suicide risk and are trained to seek adult help for themselves or friends exhibiting these signs. vs. wait-list control. | n = 1,302 ninth grade students in 16 schools in Connecticut. | Within secondary schools in Connecticut, USA. | Reduced self reported suicide attempts in intervention arm. 75% reduction in suicide planning among at risk intervention participants compared to at-risk control students. Secondary outcomes of knowledge of depression and suicide and favourable attitides to help seeking (for self and others) were improved following exposure to the SOS program. | None. | None. |
| Schonert-Reichl et al. (2015) | Enhancing cognitive and social-emotional development through a simple-to-administer mindfulness-based school program for elementary school children: a randomized controlled trial. | Randomized controlled trial; outcomes at 4 months. | SEL (mindfulness and caring for others, designed for elementary school students, would enhance cognitive control, reduce stress, promote well-being and prosociality, and produce positive school outcomes) vs. a regular social responsibility program. | n = 99; 4 classes of combined 4th and 5th graders in a western Canadian city. | Program administered in 4th and 5th grade classes. | Relative to children in the social responsibility program, children who received the SEL program with mindfulness (a) improved more in their cognitive control and stress physiology; (b) reported greater empathy, perspective-taking, emotional control, optimism, school self-concept, and mindfulness, (c) showed greater decreases in self-reported symptoms of depression and peer-rated aggression, (d) were rated by peers as more prosocial, and (e) increased in peer acceptance (or sociometric popularity). The results of this investigation suggest the promise of this SEL intervention and address a lacuna in the scientific literature-identifying strategies not only to ameliorate children's problems but also to cultivate their well-being and thriving. | None. | None. |
| Sharpe et al. (2017) | Use, acceptability and impact of booklets designed to support mental health self-management and help seeking in schools: results of a large randomised controlled trial in England. | Hierarchical cluster randomized control trial with a 2 × 2 factorial design; outcome at 1 year post intervention. | Both Targeted Mental Health in Schools (TaMHS) and booklets (TaMHS + booklets, 162 schools) vs. just TaMHS (TaMHS only, 162 schools) vs. just booklets (Booklets only, 76 schools) vs. neither TaMHS nor booklets (No intervention, 77 schools). | n = 14,690; n = 8139 primary school participants. 486 schools randomly allocated. n = 6,551 secondary school students in England LA's (local authoritiy); ~50% female, ~77% white, ~20% low SES in UK. | Primary and secondary schools. | There was no discernable impact of the booklets on mental health, quality of life or help seeking. | None. | None. |
| Shoshani et al. (2016) | Effects of the Maytiv positive psychology school program on early adolescents' well-being, engagement, and achievement. | Longitudinal study; outcomes over 2 years. | Positive psychology-based classroom-level intervention vs. waiting list control over 2 years. | n = 2,517 seventh- to ninth-grade students in 70 classrooms, from six schools in the center of Israel. | Classrooms administering school intervention program in Israel. | Positive intervention effects on positive emotions, peer relations, emotional engagement in school, cognitive engagement. | Positive interventon effects on grade point average scores among intervention group. | None. |
| Silverstone et al. (2015) | Initial Findings from a Novel School-Based Program, EMPATHY, Which May Help Reduce Depression and Suicidality in Youth. | Within subjects design; outcomes at 12 weeks post intervention. | Participant in Empowering a Multimodal Pathway Towards Healthy Youth (EMPATHY) program vs. participant who completed both assesments of the (EMPATHY) program. | n = 2,790 youth, aged 11-18 (Grades 6-12) in Alberta, Canada. | School (grades 6-12), primary health care, specialist mental health care, social services, as well as others involved with youth (such as the police services). | Results from the 2,790 students who completed scales at both baseline and 12-week follow-up showed significant decreases in depression and suicidality. Importantly, there was a marked decrease in the number of students who were actively suicidal (from n=125 at baseline to n=30 at 12-weeks). Of the 503 students offered the CBT program 163 (32%) took part, and this group had significantly lower depression scores compared to those who didn't take part. There were no improvements in self-esteem, quality-of-life, or the number of students using DAT. Only 60 students (2% of total screened) required external referral during the 24-weeks following study initiation. These results suggest that a multimodal school-based program may provide an effective and pragmatic approach to help reduce youth depression and suicidality. Further research is required to determine longer-term efficacy, reproducibility, and key program. | None. | None. |
| Skryabina et al. (2016) | Effect of a universal anxiety prevention programme (FRIENDS) on children's academic performance: results from a randomised controlled trial. | Randomized controlled trial; outcomes at 12 months post intervention. | Universal school-based cognitive behaviour therapy prevention program, FRIENDS, delivered by health care staff or school staff vs. usual personal, social, health and education (PSHE) lessons. | n = 1,343 primary school children in England. | Schools in England involving school staff or care staff along with intervention curriculum. | Found a significant reduction in self-reported anxiety (social anxiety, GAD, total anxiety) when FRIENDS delivered by health leaders external to schools. | No effect found on academic perforamance of children 12 months after participating. | None. |
| Smith et al. (2015) | Computerised CBT for depressed adolescents: Randomised controlled trial. | Randomized controlled trial; outcomes at 3 and 6 months post intervention. | Computerized cognitive behavorial therapy vs. waiting-list control. | n = 112; young people (aged 12-16) with significant symptoms of depression using multiple-informants (adolescents, parents, teachers) in South London, England. | Middle school and high school intevention program delivered via computer. | Relative to being on a Waiting List, C-CBT was associated with statistically significant and clinically meaningful improvements in symptoms of depression and anxiety according to adolescent self-report; and with a trend towards improvements in depression and anxiety according to parent-report. Improvements were maintained at follow-up. Treatment gains were similar for boys and girls across the participating age range. Treatment effect was partially mediated by changes in ruminative thinking. Teachers rated adolescents as having few emotional or behavioural problems, both before and after intervention. C-CBT had no detectable effect on academic attainment. C-CBT shows considerable promise for the treatment of mild-moderate depression in adolescents. | In the month after intervention, young people who received C-CBT had significantly fewer absences from school than those on the waiting list. | None. |
| Spoth et al. (2015) | PROSPER partnership delivery system: Effects on adolescent conduct problem behavior outcomes through 6.5 years past baseline. | Cluster randomized trial; outcomes over 6.5 years. | PROSPER(PROmoting School-community-university Partnerships to Enhance Resilience) is a universal, public health oriented approach that focuses on community-based collaboration and capacity building vs. a control condition. | n = 28 community school districts (15% of students eligible for free or reduced-cost school lunches; districts were located in communities having populations ranging from approximately 7,000 to 45,000. Student participants were in the 6th grade at the time of study pretesting). | Family-focused school-delivered intervention. | None. | Findings suggest that the PROSPER delivery system has the potential to reduce adolescent coduct problems behaviors (CPBs) in general populations. | None. |
| Taylor et al. (2015) | A qualitative process evaluation of a randomised controlled trial of a parenting intervention in community (school) settings for children at risk of attention deficit hyperactivity disorder (ADHD). | Qualitative process evaluation of feasibilty and acceptability of intervention tested in RCT. | A school-based parenting intervention program for parents and teachers of children with high levels of ADHD symptoms- qualitative evaluation of feasibilty and acceptability. | n = 51. Parents (n = 22) and teaching staff (n = 29) of children with high levels of ADHD symptoms. | Participants sampled from within schools in the midlands in the UK and parenting intervention delivered in school setttings. | No mental health outcomes. | None. | The parenting intervention was acceptable to parents and teachers. Differential attitudes were elicited with parents generally stating a preference for universal recruitment approaches and teachers describing the need to target specific parents. |
| Townsend et al. (2017) | **The Association of School Climate, Depression Literacy, and Mental Health Stigma Among High School Students.** | Randomized controlled trial; outcomes after one academic school year. | Maryland Safe Supportive Schools Project vs. a randomized controlled trial of the Adolescent Depression Awareness Program. | n = 2,386 students in 9th and 10th grade in 5 high schools in Maryland. | 5 private Catholic high schools in Maryland. | None. | Positive school climate was associated with greater odds of depression literacy and endorsement of fewer stigmatizing beliefs among students. Authors suggest findings indicate aspects of school environment may facilitate or inhibit students' recognition of depression and subsequent treatment-seeking. | None. |
| Vekas et al. (2017) | The impact of a universal intervention targeting perfectionism in children: An exploratory controlled trial. | Quasi-experiment; outcomes at 3 months post intervention. | Three‐lesson perfectionism intervention (cognitive behavioural therapy) with children vs. control condition (no therapy). | n = 212 students completing years 5 and 6 (mean age = 11.1 years; SD = 0.60; range 10.08–12.79) in South Australia | 5 schools in south Australia; intervention delivered in classrooms. | At post-intervention, children in the intervention group had significantly lower perfectionism than the control group and at 3-month follow-up had significantly higher levels of well-being. As predicted by theory, decreases in perfectionism mediated the relationship between condition and improved well-being.This exploratory study provides evidence for the usefulness of a brief universal prevention programme targeting perfectionism. Future research should use more robust designs, explore longer-term effects, and the impact on a wider range of variables, including scholastic achievement. | None. | None. |
| Warchburger et al. (2018) | The Efficacy of a Universal School-Based Prevention Program for Eating Disorders among German Adolescents: Results from a Randomized-Controlled Trial. | Cluster-randomized design; outcomes at 3 and 12 months post intervention. | POPS-program (POtsdam Prevention at Schools), a universal school-based eating disorder prevention program vs. waiting control group. | n = 1,112 adolescents aged 10 to 16 years participated (49% girls; 51% intervention group) in Germany. | Schools in Germany; intervention delivered in classrooms. | The intervention group showed a more favorable course compared to the control group regarding all observed risk factors for eating disorders except for perceived teasing. Effect sizes were small but comparable to other primary prevention programs. At 1-year follow-up, a small but significant effect on disordered eating was observed. Results of the per-protocol analyses were mostly confirmed by the intention-to-treat analyses. Results were promising for both genders although girls benefited more regarding disordered eating and internalization of the thin ideal. Further studies are warranted examining successful program elements and whether gender-specific programs are needed. | None. | None. |
| Wasserman et al. (2015) | School-based suicide prevention programmes: the SEYLE cluster-randomised, controlled trial. | Cluster-randomized controlled trial; outcomes at 3 and 12 months. | Comparison of three suicide prevention programs: (1) Question, Persuade, and Refer (QPR), a gatekeeper training module targeting teachers and other school personnel, (2) the Youth Aware of Mental Health Programme (YAM) targeting pupils, and (3) screening by professionals (ProfScreen) with referral of at-risk pupils. | n = 11,110 adolescent pupils, median age 15 years (IQR 14–15), recruited from n = 168 schools in ten European Union countries. | Within European elementary schools. | Primary outcome measure was the number of suicide attempt(s) made by 3 month and 12 month follow-up. No significant group differences at 3 months. At 12 months, YAM was associated with a significant reduction of incident suicide attempts ( [OR] 0·45, 95% CI 0·24–0·85; p=0·014) and severe suicidal ideation (0·50, 0·27–0·92; p=0·025), compared with the control group. | None. | None. |
| Wing et al. (2015) | A school-based sleep education program for adolescents: a cluster randomized trial. | Cluster randomized controlled trial; outcomes at 5 weeks after the intervention. | Sleep education program (Healthy Sleep, Healthy School Life: 1 hour town hall seminar, two 40-minute small class workshops held once per month, a slogan competition, a brochure, an educational Web site and parents and teachers offered sleep education seminars) vs. schools which did not receive any sleep program. | n = 3,713 students (intervention: 1545 vs control: 2168; 40.2% boys; mean age ± SD: 14.72 ± 1.53 years) in Hong Kong. | 14 secondary schools in Hong Kong; included a town hall seminar, small class workshops, a slogan competition, a brochure, and an educational web site. | The students in the intervention group had significantly improved sleep knowledge compared with the control group (mean difference: 3.64 [95% confidence interval (CI): 3.21 to 4.07]; Cohen's d = 0.51) as measured by using a sleep knowledge questionnaire. Weekday sleep duration was reduced in both groups, and the significant difference in weekday sleep duration was lost in the intention-to-treat analysis (mean difference: 0:01 [95% CI: -0:00 to 0:04]). In addition, the intervention group had a lower incidence of consuming caffeine-containing energy drinks (adjusted odds ratio: 0.46 [95% CI: 0.22 to 0.99]) and had better behavioral (mean difference: -0.56 [95% CI: -1.02 to-0.10]; Cohen's d = 0.13) and mental health (mean difference: -0.30 [95% CI:-0.15 to -0.46]; Cohen's d = 0.11) outcomes. A school-based sleep education program was effective in enhancing sleep knowledge and improving behavioral and mental health, but it had no significant impact on sleep duration or pattern among adolescents. | A school-based sleep education program was effective in enhancing sleep knowledge and improving behavioral and mental health, but it had no significant impact on sleep duration or pattern among adolescents. | None. |

| **Community Interventions: Homeless Services** | | | | |  |  |  |  |
| --- | --- | --- | --- | --- | --- | --- | --- | --- |
| **Author** | **Title** | **Design** | **Interventions** | **Participants** | **Community Setting/Involvement** | **Mental Health Outcomes** | **Individual or Community-level Social Outcomes** | **Other Outcomes** |
| Aubry et al. (2015) | One-year outcomes of a randomized controlled trial of housing first with ACT in five Canadian cities. | Nonblind, parallel-group Randomized controlled trial (RCT); outcomes over 1 year. | Housing First (rent supplement, assistance to find housing, and assertive community treatment) vs. treatment as usual. | n = 950 high-need participants with severe mental illness, who were either absolutely homeless or precariously housed in five Canadian cities (Vancouver, Winnipeg, Toronto, Montreal, and Moncton). | Health and social service agencies. | Improvement in overall quality of life was significantly greater among Housing First participants compared with treatment-as-usual participants. | At one-year follow-up, 73% of Housing First participants and 31% of treatment-as-usual participants resided in stable housing. Housing First participants also showed greater improvements in community functioning compared with treatment-as-usual participants. | None. |
| Aubry et al. (2016) | A randomized controlled trial in five Canadian cities of the effectiveness of Housing First with assertive community treatment for persons with serious mental illness and a history of homelessness. | Randomized controlled trial; outcomes over 24 months. | Housing First program including assertive community treatment vs. usual care. | n = 950 homeless or precariously housed adults with serious mental illness living in 5 cities in Canada. | Subsidized housing units in the community. | HF participants reported higher quality of life at 2-year followup. | Compared to usual care, HF participants who entered housing did so more quickly (73 vs. 220 days), had longer housing tenures at 2-year followup (281 vs. 115 days, AAD), and rated the quality of their housing more positively. They also had higher levels of community functioning. | None. |
| Chung et al. (2018) | **Housing First for older homeless adults with mental illness: a subgroup analysis of the At Home/Chez Soi randomized controlled trial.** | Randomized controlled trial; outcomes over 24 months (subgroup analysis comparing older and younger participants). | Housing First program including assertive community treatment or intensive case management, depending on level of need vs. usual care. | n = 2,148 homeless adults with mental illness living in 5 cities in Canada (470 fifty or more years old vs. 1678 under fifty years old). | Subsidized housing units in the community. | Improvements from baseline to 24 months in mental health and condition-specific quality of life were significantly greater among older homeless adults than among younger homeless adults. | At 24 months, HF significantly improved the percentage of days stably housed among older and younger homeless adults, by 44% and 40%, respectively, as compared with usual care, with no significant differences between age groups. | None. |
| Crisanti et al. (2017) | A longitudinal analysis of peer-delivered permanent supportive housing: Impact of housing on mental and overall health in an ethnically diverse population. | Controlled trial; outcomes over 6 months. | Permanent supportive housing (PSH) vs. treatment as usual. | n = 237 persons who were homeless or at risk for homelessness who had a serious mental illness (SMI). | Apartments (obtained through PSH) in the community. | Both groups experienced decreased psychological distress after baseline. | Psychological distress across groups was associated with type of housing, and history of violence or trauma. | PSH was associated with good to excellent health 6 months after baseline, and greater than comparison group. |
| Kerman et al. (2018) | The effects of housing stability on service use among homeless adults with mental illness in a randomized controlled trial of housing first. | Randomized controlled trial; outcomes over 24 months. | Housing First (housing and support) vs. standard care. | n = 2,039 homeless persons with mental illness in five cities in Canada. | Apartments (obtained through Housing First) in the community. | Persons achieving housing stability had decrease inpatient psychiatric hospitalization, and decreased Emergency Department use. | Persons achieving housing stability had decreased food bank use, less prison time, and less homeless shelter use. | Similar mental health and social determinants outcomes seen in Housing First vs. standard care, except for prison time which was greater in the Housing First group that was unstably housed. |
| Kozloff et al. (2016) | "Housing First" for Homeless Youth With Mental Illness. | Randomized controlled trial; outcomes over 24 months. | Housing First program including assertive community treatment or intensive case management, depending on level of need vs. usual care. | n = 156 homeless youth with mental illness in 5 Canadian cities (87 participating in Housing First vs. 69 receiving usual care). | Subsidized housing units in the community. | None. | Housing First participants were stably housed a significantly greater proportion of time compared to usual care participants (65% vs. 31%). | None. |
| Nath et al. (2016) | The Impact of Drop-In Centres on the Health of Street Children in New Delhi, India. | Cross-sectional study; outcomes at 12 months. | Visits to one of two drop-in centers. | n = 134 homeless children in New Delhi, India (69 attenders and 65 nonattenders of drop-in centers). | Drop-in homeless service centers. | Attendees had better mental health outcomes than non-attendees. | None. | For every month of attendance at a drop-in center, children experienced 2.1% fewer ill health outcomes per month and used 4.6% fewer substances. Children were also less likely to have been a current substance user than a never substance user for every additional month of attendance at a center. |
| Nelson et al. (2016) | Collaboration and involvement of persons with lived experience in planning Canada's At Home/Chez Soi project. | Qualitative experiment; outcomes over 1 year. | 45 to 90 minute interviews conducted in English or French. | n = 131 local stakeholders (Site Co‐ordinators, Site Principal Investigators, PWLE and service providers who participated in the planning) in 5 Canadian cities (Vancouver, Winnipeg, Toronto, Montreal and Moncton). | Multi sector community agency collaboration. | None. | Strategies that enhanced collaboration included the development of a common vision, values and purpose around the Housing First approach, developing a sense of belonging and commitment among stakeholders, bridging strategies employed by Site Co-ordinators and multiple strategies to engage PWLE. At the same time, a tight timeline, initial tensions, questions and resistance regarding project and research parameters, and lack of experience in engaging PWLE challenged collaboration. In a hybrid planning environment, clear ommunication and specific strategies are required that flow from an understanding that the process is neither fully participatory nor expert-driven, but rather a hybrid of both. | None. |
| O'Campo et al. (2016) | How did a Housing First intervention improve health and social outcomes among homeless adults with mental illness in Toronto? Two-year outcomes from a randomised trial. | Randomized controlled trial; outcomes over 2 years. | Housing First program including assertive community treatment vs. treatment as usual (TAU). | n = 197 homeless adults with mental illness in Toronto. | Subsidized housing units in the community. | Housing First participants had greater quality of life subscale scores at some time points. | Housing First participants were stably housed a significantly greater proportion of time compared to usual care participants (46% mean difference between groups). They also had higher levels of community functioning and lower rates of arrest at some time points. | No significant differences between-group over the followup period were observed for health service usage, community integration, or substance use. |
| Poremski et al. (2016) | Effects of Housing First on Employment and Income of Homeless Individuals: Results of a Randomized Trial. | Randomized controlled trial; median followup period of 745 days. | Housing First program including assertive community treatment or intensive case management, depending on level of need vs. usual care. | n = 2,148 homeless adults with mental illness living in 5 cities in Canada. | Subsidized housing units in the community. | None. | HF did not significantly improve rates of employment or increase income, compared to usual care. | None. |
| Salize et al. (2015) | [Improving Mental Health Care in People at Risk for Getting Homeless]. | Longitudinal study; outcomes 6 months post treatment. | Patient seen/referred by social welfare services and labour agencies, and referral to community mental health services. | n = 58 people with mental health diagnoses at risk of losing housing diagnosed and motivated to initiate treatment in a community mental health service in Germany. | Social welfare services or labour agencies. | During a 6-month follow-up quality of life and social support was improved (partly statistically significant) and psycho-social needs for care decreased. 24 were referred to regular mental health care, 8 were stabilized enough after the initial motivational to refrain from acute treatment, 26 dropped out. | Motivational interviewing is likely to increase insight into illness and acceptance of mental health care in untreated persons with mental disorders at risk for social decline. | None. |
| Shinn et al. (2015) | Longitudinal Impact of a Family Critical Time Intervention on Children in High-Risk Families Experiencing Homelessness: A Randomized Trial. | Randomized trial; outcomes over 3, 9, 15, and 24 months. | FCTI (combines housing and structured, time-limited case management to connect families leaving shelter with community services) vs. usual care. | Children in 200 newly homeless families in which mothers had diagnosable mental illness or substance problems in Westchest County, NY. | Homeless shelter system. | Both experimental and control children in all age groups showed reductions in symptoms over time (mental health, psychosocial symptomjs). Although experimental results were scattered, they suggest that FCTI has the potential to improve mental health and school outcomes for children experiencing homelessness. | The intervention led to declines in self-reported school troubles for children aged 6-10 and 11-16. | None. |
| Stergiopoulos et al. (2015) | Effect of scattered-site housing using rent supplements and intensive case management on housing stability among homeless adults with mental illness: a randomized trial. | Unblinded, randomized trial; outcomes over 2 years. | Scattered-site housing (using rent supplements) and off-site Intensive Care Management (ICM) services vs. usual care group had access to existing housing and support services in their communities. | n = 1198 homeless adults with mental illness who do not require the treatment intensity of Assertive Community Treatment in 4 Canadian Cities (Vancouver, Winnipeg, Toronto, and Montreal). | Communiy agencies and institutions serving homeless individuals, including shelters, drop-in centers, criminal justice programs, and hospitals. | The mean change in quality of life EQ-5D score from baseline to 24 months among the intervention group was not statistically different from the usual care group. | The adjusted percentage of days stably housed was higher among the intervention group than the usual care group. | None. |
| Stergiopoulos et al. (2016) | The effectiveness of a Housing First adaptation for ethnic minority groups: findings of a pragmatic randomized controlled trial. | Unblinded, randomized pragmatic field trial; outcomes over 24 months. | Housing First program using rent supplements and intensive case management, enhanced by anti-racism and anti-oppression practices for homeless adults with mental illness from diverse ethnic minority backgrounds vs. usual care. | n = 237 adults in Toronto from ethnic minority groups experiencing mental illness and homelessness (135 participating in HF vs. 102 receiving usual care). | Subsidized housing units in the community and case manager assistance with linkage to community resources. | No significant difference between intervention and usual care on mental health symptomatology measured by Colorado Symptom Index. | Adapted HF participants were stably housed a significantly greater proportion of time compared to usual care participants (75% vs. 41%). They also had higher levels of community functioning as measured by Multnomah Community Ability Scale at 2-year followup. | No significant differences were found at 2-year followup in health status, self-reported hospital days, number of ER visits, quality of life, substance use severity, or number of arrests. Baseline diagnosis of psychosis was associated with reduced likelihood of being housed for at least half of the study period. |
| Urbanoski et al. (2018) | **Effects of comorbid substance use disorders on outcomes in a Housing First intervention for homeless people with mental illness.** | Randomized controlled trial; outcomes over 24 months. | Housing First (HF) with Intensive Case Management or Assertive Community Treatment vs. Treatment as Usual (TAU), focused on persons with substance use disorders (SUD) and those without SUD in both groups. | n = 2,154 persons with homelessness and mental disorders in Canada. | Apartments (obtained through Housing First) in the community. | There was no difference between those with and without SUD in the effects of HF (over TAU) on mental health symptoms. | Persons with SUD in HF and TAU groups spent less time in stable housing, but effects of HF did not vary by SUD stays. | No difference between those with and without SUD in the effects of HF (over TAU) on quality of life, community functioning, health-related quality of life. |

| **Community Interventions: Criminal Justice** | | | |  |  |  |  |  |
| --- | --- | --- | --- | --- | --- | --- | --- | --- |
| **Author** | **Title** | **Design** | **Interventions** | **Participants** | **Community Setting/Involvement** | **Mental Health Outcomes** | **Individual or Community-level Social Outcomes** | **Other Outcomes** |
| Abracen et al. (2016) | Individual Community-Based Treatment of Offenders With Mental Illness: Relationship to Recidivism. | Retrospective chart reviews; outcomes over 1 year. | Treatment provided in detention facility; 86 offenders received no treatment at all vs. 23 offenders received “moderate” level of treatment (19 or fewer individual therapy sessions) vs. 27 offenders received a “high” level of treatment (20 or more individual therapy sessions). | n = 136 high-risk, high-need federal offenders with a variety of mental health diagnoses, housed in a Community Correctional Centre in a metropolitan area in Canada. | Community Correctional Centre in Ontario Canada, considered a minimum-security institution. | None. | Decrease in likelihood of recidivism proportional to amount of mental health treatment received. | None. |
| Batastini et al. (2016) | Connecting the disconnected: Preliminary results and lessons learned from a telepsychology initiative with special management inmates. | Randomized control trial; outcomes after 6 weeks. | Treatment provided in detention facility; telepsychology therapy vs. in-person therapy vs. no-treatment control over 6 weeks. | n = 49 high-security, administratively segregated inmates in the Kansas Department of Corrections. | High-security prison with administratively segregated inmates in Kansas. | None. | Although some evidence indicated that telepsychology was less preferred than in-person sessions, group differences on measures of psychological functioning and criminal thinking were not found across 3 conditions (telepsychology, in-person, and a no-treatment control). | None. |
| Clark et al. (2016) | Evaluation of the Impact Personality Disorder Project - A psychologically-informed consultation, training and mental health collaboration approach to probation offender management. | Descriptive study; outcomes at 1, 2, and 3 years post-release. | Camden and Islington Impact Personality Disorder Project provided psychologically informed consultation and training to probation staff in a Public Protection Unit and staff working in 2 probation hostels, working with high-risk prison sentenced offenders on licence (probation) in the community. | 10 probation officers each carrying a caseload of 40-50 probationers deemed high-risk (many but not all with a personality disorder diagnosis) in the UK. | London probation office and 2 probation hostels. | None. | Decrease in rate of recalls to prison from probation in the first year of the intervention, sustained over the second and third year. There was no change in re‐arrest or new charge rates. | None. |
| Compton et al. (2016) | Opening Doors to Recovery: Recidivism and Recovery Among Persons With Serious Mental Illnesses and Repeated Hospitalizations. | Quantitative study; outcomes over 4, 8, and 12 months. | Opening Doors to Recovery, an intensive, team-based community support program for persons with mental illness and a history of inpatient psychiatric recidivism. | n = 100 persons with mental illness and a history of inpatient psychiatric recidivism in southeast Georgia. | Hospital, crisis stabilization unit, participant's home, or community settings. | Hospitalizations decreased, from 1.9±1.6 to .6±.9 (p<.001), as did hospital days, from 27.6±36.4 to 14.9±41.3 (p<.001). Significant linear trends were observed for recovery measures, and trajectories of improvement were apparent across the entire follow-up period. | None. | None. |
| Compton et al. (2017) | A potential new form of jail diversion and reconnection to mental health services: I. Stakeholders' views on acceptability. | Qualitative study; outcomes over 2 years. | Sequential Intercept Model Intercept 1, during the police-patient encounter, aiming to intervene at the pre‐booking diversion level; focus groups conducted with CIT-trained and non-CIT-trained officers. | n = 49 individuals with serious mental illnesses receiving treatment who also have a prior criminal justice history; 9 focus groups. | Trained CIT police officers serve as specialized, first‐line responders for calls involving persons in crisis; non‐CIT‐trained officers; Mental health and criminal justice systems in the state of Georgia. | Officers often perceived themselves to be the frontline mental health providers, despite little training in offering mental health care, that most seem genuinely invested in helping patients, and that the primary concern they face is often having no suitable disposition for people in crisis other than hospital or jail. | Focus groups revealed that patients enrolled with the hope that the linkage system would prevent negative interactions with police and minimize risk of arrest. Officers reported preferring not to arrest mental health patients and were genuinely invested in helping them, and felt that the linkage system might be an additional tool during encounters. | None. |
| Compton et al. (2017) | A potential new form of jail diversion and reconnection to mental healthservices: II. Demonstration of feasibility. | Feasibility study; outcomes at 12 months. | Sequential Intercept Model Intercept 1, during the officer–patient encounter, aiming to intervene at the pre‐booking diversion level. | n = 206 eligible individuals with serious mental illnesses and an arrest history. | Georgia's community service boards (CSBs), which are 26 quasi‐governmental mental health agencies that serve as the safety net for the provision of mental health services across the state. | Number of arrests decreased for individuals with serious mental illnesses. | Mental health services lack coordination and communication between the mental health and criminal justice systems. Here, researchers observed potential effectiveness of the linkage system in reducing the number of incarcerations in the year of enrollment in the system, as compared with the previous year. | None. |
| Ellison et al. (2015) | Adapting supported employment for emerging adults with serious mental health conditions. | Open trial feasibility study; outcomes through 12 months. | Sequential Intercept Model Intercept 4, re-entry; Individual Placement and Support (IPS) model of supported employment adapted for a psychiatric treatment program for early-emerging adults. | n = 35 participants aged 17-20 years, primarily African American, under the custody of the state, with a primary mood disorder diagnosis. | Thresholds Young Adult Program (YAP), a specialized residential treatment program that provides comprehensive clinical services (i.e., psychiatry, individual and group therapy, and intensive case management), vocational and transitional services, and community housing young adults (aged 16-21) with at least one Axis-1 DSM-IV diagnosis. Illinois Department of Children and Family Services and Division of Mental Health. | None. | 80% retention rate; 49% of participants started a job and/or enrolled in an education program. | None. |
| Frisman et al. (2017) | Evaluation of CT's ASIST program: Specialized services to divert higher risk defendants. | Quasi-experiment with linked administrative data; outcomes at 6 months post intervention. | Sequential Intercept Model Intercept 2 and 3, pre-sentencing (initial hearing/detention) or post-sentencing (jail, court) intercepts; Advanced Supervision and Intervention Support Team (ASIST), which offers criminal justice supervision in conjunction with mental health treatment and support services to address criminogenic needs. | >500 clients, arraignment court defendants with SMI who are deemed unlikely to succeed in regulardiversion programs, as well as persons with moderate to serious mental illness who are probationers, parolees, or clients of Alternative in the Community centers (pre-trial and probation clients). | Nine ASIST service locations in central and southern Connecticut; program developed by a state-level interagency workgroup comprising mental health, behavioral health, correctional department, and court leaders. | Clients showed improvements in mental health. | There was no change in arrest rates, but a significant reduction in re-incarceration. | None. |
| Grabert et al. (2017) | Expedited Medicaid Enrollment, Service Use, and Recidivism at 36 Months Among Released Prisoners With Severe Mental Illness. | Quasi-experiment; outcomes over 36 months. | Sequential Intercept Model Intercept 4; prison releasees referred to expedited Medicaid enrollment vs. control group (not referred). | n >3000 prison releasees with severe mental illness (schizophrenia or bipolar disorder) in Washington State. | Medicaid, the Department of Social Health Services (general mental health service use), and the Department of Corrections in Washington State. | Expedited Medicaid enrollment had a sustained effect on increased use of community mental health and general medical services (p<.01) 36 months after prison release. | Expedited Medicaid enrollment had a sustained effect on increased months of enrollment (p<.01) 36 months after prison release. However, expedited Medicaid enrollment did not reduce criminal recidivism, consistent with 12-month findings. | None. |
| Kendall et al. (2017) | A Two-Week Psychosocial Intervention Reduces Future Aggression and Incarceration in Clinically Aggressive Juvenile Offenders. | Randomized controlled trial; outcomes at 6 and 12 months. | 2-week intervention targeting psychosocial factors implicated in risky behavior (e.g., learning strategies to manage "hot" emotions that prompt risk taking) vs. an equally intensive health promotion control. | n = 310 juveniles (13-17 years old), 90% Af-Am, 66% male, youth were recruited from evening reporting centers (ERCs) throughout Cook County, Chicago. | Evening reporting centers (ERCs): community-based alternatives to detention following arrest and offer single-sex, on-site, after- school supervision. Minors were sentenced to ERCs for 5 to 28 days in lieu of detention. | Reductions in clinical aggression and incarcerations in treatment group. | None. | None. |
| Khalsa et al. (2017) | Specialized Police-Based Mental Health Crisis Response: The First 10 Years of Colorado's Crisis Intervention Team Implementation. | Quantitative study; outcomes at 10 years. | The Memphis crisis intervention team (CIT) model (3–5), mobile crisis teams, and specialized programs such as Mental Health First Aid provide de-escalation skills and mental health literacy to increase diversion to psychiatric treatment. | CIT-trained officers; N = 6,353 reported incidents: men (n = 3,331) and women (n = 2,995). | Law enforcement agencies in Colorado. | In most incidents (N=3,964, 62.4%), at least one psychiatric diagnosis was reported. Alcohol or drug abuse was prevalent (N=2,226, 38.6%) | Incidents involving individuals in crisis who had a psychiatric illness, threatened or attempted suicide, abused substances, threatened violence, or had a weapon were more likely to be diverted to treatment, whereas violence threat, presence of a lethal weapon and substance use increased, and suicide threat decreased, the likelihood of transfer to jail. | None. |
| Knudsen et al. (2016) | A Specialized Treatment Court for Veterans with Trauma Exposure: Implications for the Field. | Program evaluation; outcomes assessed by interviews at baseline, 6, and 12 months. | Sequential Intercept Model Intercept 3, specialized court; enrollment in a jail diversion and trauma recovery Veterans Treatment Court program (no control group). | n = 86 trauma-affected veterans enrolled in a jail diversion and trauma recovery Veterans Treatment Court program. | Criminal justice system and the Ohio Department of Mental Health and Addiction Services in Columbus, Ohio. | Improvement in PTSD, depression, substance abuse, overall functioning, emotional wellbeing, relationships with others, recovery status, social connectedness, family functioning, and sleep at 6 months, sustained at 12 months. | Housing status and recidivism were examined (no baseline or control group for comparison). Throughout the 12 month study period 57 % saw no change in housing, 11.6 % transitioned from stable to unstable housing, and 31.4 % improved from unstable to stable housing. Homelessness, employment and school enrollment remained unchanged. | None. |
| Lamberti et al. (2017) | A Randomized Controlled Trial of the Rochester Forensic Assertive Community Treatment Model. | Randomized Controlled Trial; outcomes over 1 year. | Sequential Intercept Model Intercept 3, post-sentencing conditional discharge; forensic assertive community treatment (FACT) vs. enhanced treatment as usual for one year. | n = 70 adults with psychotic disorders who were arrested for misdemeanor crimes and who were eligible for conditional discharge were recruited from the Monroe County, New York, court system. | Care delivered in community locations in accordance with Assertive Community Treatment model of care; weekly meetings between a FACT team liaison, the presiding judge, and representatives from the Public Defender and District Attorney offices. | More time in outpatient mental health and less time in the hospitals. | Rochester FACT model was associated with fewer convictions for new crimes, less time in jail and hospitals, among justice-involved adults with psychotic disorders compared with treatment as usual. | None. |
| Lloyd-Evans et al. (2017) | National implementation of a mental health service model: A survey of Crisis Resolution Teams in England. | Quasi experiment with additional focus groups. | Crisis resolution teams (acute home treatment services). | n = 192 of Crisis Resolution Teams (CRT) managers in England. | n = 218 crisis resolution teams in 65 mental health NHS trusts in England (nurses were in all CRT teams; two-thirds of CRTs were co-located with mental health inpatient unit and one-third on the same site as community mental health services). Individuals in CRT teams may include psychiatrists, nurses, social workers, occupational therapists, psychologists, and/ or pharmacists | None. | Almost no teams adhered fully to government implementation guidance. CRT managers identified several aspects of CRT service delivery as desirable but not routinely provided. A national policy mandate and government guidance and standards have proved insufficient to ensure CRT implementation as planned. This survey supports a survey conducted last year where only 14% of patients felt they received the right help from mental health services (Care Quality Commission, 2015). | None. |
| McKenna et al. (2015) | A prison mental health in-reach model informed by assertive community treatment principles: evaluation of its impact on planning during the pre-release period, community mental health service engagement and reoffending. | Naturalistic study; outcomes assessed at 12 months. | Treatment provided in detention facility; assertive community treatment-informed prison in-reach model of care (PMOC) vs. treatment as usual. | n = 170 prisoners with SMI released from four prisons compared with 180 such individuals released the year prior to program implementation in New Zealand. | Four prisons in the New Zealand Department of Corrections. | Increase in contacts with community mental health services, both pre- and post-release. | Increase in contacts with some social care agencies in some prisons. | None. |
| Morrissey et al. (2016) | Expedited Medicaid Enrollment, Mental Health Service Use, and Criminal Recidivism Among Released Prisoners With Severe Mental Illness. | Quasi-experiment with linked administrative data; outcomes over 12 months. | Sequential Intercept Model Intercept 4; prison releasees referred to expedited Medicaid enrollment vs. control group (not referred). | n = 3,086 released prisoners with a severe mental illness (schizophrenia or bipolar disorder) in Washington State. | Washington State state prisons, expedited Medicaid program, and Community Service Officers. The linked administrative data was provided from the Washington State Department of Social and Health Services (DSHS) in the state of Washington. | Referral for expedited Medicaid enrollment on release from prison greatly increased Medicaid enrollment (p<.01) and use of community mental health and general medical services (p<.01) for persons with severe mental illness. | No evidence was found that expediting Medicaid enrollment reduced criminal recidivism. | None. |
| Sorensen et al. (2016) | How Does the Fast Track Intervention Prevent Adverse Outcomes in Young Adulthood? | Randomized controlled trial; outcomes over 15 years. | Fast Track (provide children academic tutoring and lessons in social skills and self-control) vs. control group. | n = 891 high-risk children in four communities (Durham, North Carolina; Nashville, Tennessee; rural Pennsylvania; and Seattle, Washington). | The intervention in elementary school included: teacher-led curriculum; parent training groups; home visits; child social-skill training groups; tutoring for children in reading; and peer pairing to enhance children’s friendships in the classroom. The adolescent phase (Grades 6–10) included both standard and individualized activities for youth and families. | Fast Track proved less valuable for the prevention of mental and physical health problems. | A decomposition of treatment effects indicates that about a third of Fast Track’s impact on later crime outcomes can be accounted for by improvements in social and self-regulation skills during childhood (ages 6–11), such as prosocial behavior, emotion regulation and problem solving. | None. |
| Stewart et al. (2017) | The impact of a community mental health initiative on outcomes for offenders with a serious mental disorder. | Survival analyses; outcomes at 3 and 6 months as well as 2 and 4 years. | Sequential Intercept Model Intercept 4, re-entry; enhanced clinical discharge planning (CDP) services vs. community mental health-specialized services (CMHS) vs. CDP + CMHS vs. no community mental health initiative services. | n = 646 male federal prisoners who also have at least one major mental disorder. | Community mental health services in Canada. | None. | A significantly lower risk of returning to custody and of recidivism. The advantages were apparent within 3-6 months and sustained for up to 4 years. | None. |

| **Community Interventions: Global Mental Health** | | | |  |  |  |  |  |
| --- | --- | --- | --- | --- | --- | --- | --- | --- |
| **Author** | **Title** | **Design** | **Interventions** | **Participants** | **Community Setting/Involvement** | **Mental Health Outcomes** | **Individual or Community-level Social Outcomes** | **Other Outcomes** |
| Bass et al. (2016) | A Randomized Controlled Trial of a Trauma-Informed Support, Skills, and Psychoeducation Intervention for Survivors of Torture and Related Trauma in Kurdistan, Northern Iraq. | Randomized controlled trial; outcomes at 1 month. | 6-12 counseling sessions (trauma-informed support, skills, and psychoeducation) held by community mental health workers vs. waitlist control for about 1 month. | n = 209 with trauma exposure, psychological distress, and functional impairment in Iraq. | Community mental health clinics in northern region of Iraq. | The supportive counseling program had statistically and clinically significant impacts on the primary outcomes of depression and dysfunction and significant but smaller impacts on anxiety. | This study was carried out in a zone with periodic violence. | None. |
| Chibanda et al. (2016) | Effect of a Primary Care-Based Psychological Intervention on Symptoms of Common Mental Disorders in Zimbabwe: A Randomized Clinical Trial. | Cluster-randomized clinical trial; outcomes at 6 months. | Lay health workers administered 6 problem solving therapy sessions (plus optional 6 session peer support group) vs. enhanced standard care for 6 months. | n = 573 participants screened positive for CMD (Common mental disorder) symptoms. | 24 city health primary care clinics in Zimbabwe. | CMD symptoms decreased among intervention arm participants. | Peer support meetings were integrated into clinic activities after the study showed benefit of peer support. | None. |
| Cilliers et al. (2016) | Reconciling after civil conflict increases social capital but decreases individual well-being. | Randomized control trial; outcomes at 9 and 31 months. | Fambul Tok (community level reconciliation forums) treatment group vs. control group. | n = 2,383 individuals from 200 in Sierra Leone | 200 villages in Sierra Leone | Worsened psychological health, increasing depression, anxiety, and posttraumatic stress disorder in these same villages. | Greater forgiveness of perpetrators and strengthened social capital: Social networks were larger, and people contributed more to public goods in treated villages. | None. |
| Cluver et al. (2016) | Reducing child abuse amongst adolescents in low- and middle-income countries: A pre-post trial in South Africa. | Pre-post study; outcomes at 2 to 6 weeks post intervention. | Parenting program facilitated by local NGO childcare workers for 12 weeks. | n = 230 participants, including youth and their caregivers in South Africa. No exclusion criteria. | Parenting program was held in local church halls or other public location in 6 rural and peri-urban communities in Eastern Cape South Africa; programming and research took place in close partnership with the Provincial and National Departments of Social Development and Education and UNICEF. | Reductions reported by adolescents and caregivers in adolescent aggressive behavior. Secondary outcomes showed reduced parental and adolescent depression and caregiver substance use. | Reductions reported by adolescents and caregivers in child abuse, poor monitoring/inconsistent discipline, adolescent deliquency, and improvements in positive/involved parenting. Secondary outcomes showed improved social support and reduced parenting stress. | None. |
| Khan et al. (2017) | Evaluating feasibility and acceptability of a local psycho-educational intervention for pregnant women with common mental problems affected by armed conflict in Swat, Pakistan: A parallel randomized controlled feasibility trial. | Parallel randomized controlled feasability trial (2-arm randomized controlled feasibility trial and qualitative evaluation of the acceptability of the psycho-educational intervention); outcomes at 2 months post intervention. | 2 psycho-educational sessions administered by community health workers vs. routine care. | n = 81 pregnant women with SRQ score of ≥9 in Swat, Pakistan. | Community health worker psycho-educational sessions delivered in women's homes. | Primary outcome was help-seeking for psychological distress by pregnant women at 2 months after the intervention - the outcome evaluation was not powered but more women in the intervention arm compared to control arm sought assistance for distress. | At follow-up 2 months post-intervention, there was no difference between the intervention and control arms in the levels of perceived social support or severity of psychological distress symptoms. | None. |
| Munetsi et al. (2018) | Trained lay health workers reduce common mental disorder symptoms of adults with suicidal ideation in Zimbabwe: a cohort study. | Secondary analysis of a cluster-randomized clinical trial; outcomes at 6 months. | Lay health workers administered 6 problem solving therapy sessions (plus optional 6 session peer support group) vs. enhanced standard care for 6 months. | n = 573 participants screened positive for CMD (Common mental disorder) symptoms. | 24 city health primary care clinics in Zimbabwe. | CMD symptoms significantly decreased among intervention arm participants; this difference was similar among those with suicidal ideation (SI) at baseline and those who did not. | At baseline, participants from households without an income had 2x the odds of suicidal ideation (SI) compared to those from households with an income, and participants with insufficient food had nearly 3x the odds of SI. Widowed, divorced, separated individuals had higher risk of SI. | None. |
| Overbeek et al. (2015) | Mediators and Treatment Factors in Intervention for Children Exposed to Interparental Violence. | Randomized controlled trial; outcomes at 1 week, and 6 months post intervention. | Nine 90-minute sessions for interpersonal violence-focused (IPV) vs. common factors community-based group intervention. | N = 134 dyads (children 6-12 year olds and their caretaking parent) in the Netherlands. | Children and parents in community-based group intervention. | Children in the IPV intervention were less likely to have clinical levels of PTS symptoms than children in the common factors intervention (B = −0.56, t(1, 274.92) = −3.80, p < .001). The level of mean coping skills was higher in the IPV-intervention condition than in the common factors condition. | None. | None. |
| Patel et al. (2017) | The Healthy Activity Program (HAP), a lay counsellor-delivered brief psychological treatment for severe depression, in primary care in India: a randomised controlled trial. | Randomized controlled trial; outcomes at 3 months. | Healthy Activity Program (HAP): lay counselors conducted 6-8 sessions (behavioral activation as the core psychological framework with added emphasis on strategies such as problem-solving and activation of social networks) vs. enhanced usual care delivered by lay counsellors for 3 months. | n = 495 participants screening more than 14 on PHQ9 in India. | 10 primary care centers in Goa, India. | Intervention group had strong effect on depression remission and depression symptom severity at 3 months. Secondary outcomes also showed improvement in intervention group (disability score, days unable to work, behavioral activation score, SI, intimate partner physical violence experienced by women) however there was no change in intimate partner psychological/emotional violence experienced by men or women or intimate partner physical violence experienced by men. | Days unable to work in past 1 month are lower in the intervention group. Women who received HAP were nearly 50% less likely to report intimate partner physical violence at the end of treatment than were women in usual care. | None. |
| Rahman et al. (2016) | Effect of a Multicomponent Behavioral Intervention in Adults Impaired by Psychological Distress in a Conflict-Affected Area of Pakistan: A Randomized Clinical Trial. | Randomized clinical trial; outcomes at 3 months post intervention. | Lay health workers administered 5 weekly 90-minute session (empirically supported strategies of problem solving, behavioral activation, strengthening social support, and stress management) vs. enhanced standard care for 3 months. | n = 346 participants with high levels of psychological distress and functional impairment in Pakistan. | 3 primary care centers in Peshawar, Pakistan. | Clinically significant reduction in anxiety and depressive symptoms at 3 months. | This study was carried out in a conflict zone with ongoing bomb blasts and attacks. Despite the ongoing violence, the intervention showed a decrease in primary outcomes (anxiety, depressive symptoms). In addition, it tracked whether there was a decrease in the problems for which the person sought help (not described in the article as these are open ended) which could include social determinants of health such as safety, support systems, stress. | None. |
| Van Andel et al. (2016) | Optimizing foster family placement for infants and toddlers: A randomized controlled trial on the effect of the foster family intervention. | Randomized controlled trial; outcomes at 6 months post intervention. | Foster care workers conducted six 90-minute home visits providing (background) information on interactional and attachment themes in starting relationships vs. standard care visits every 2 to 6 weeks. | n = 123 foster care children in the Netherlands. | 12 foster care organizations. | The study showed that this intervention has a positive effect on parenting skills which may in turn help the child and foster parents build secure relationships. | None. | None. |
| Weiss et al. (2015) | Community-based mental health treatments for survivors of torture and militant attacks in Southern Iraq: a randomized control trial. | Randomized control trial; outcome over 3.5 months. | Transdiagnostic counseling intervention (CETA) in one site and cognitive processing therapy (CPT) in a second site, with separate wait-list controlls in both sites. | n = 343; elevated trauma symptoms and experience of systematic violence in Southern Iraq. | Ministry of Health primary health care centers or client's homes, 2 areas of Southern Iraq. | The CETA intervention showed large effect sizes for all outcomes (trauma symptoms and dysfunction, depression and anxiety symptoms). The CPT intervention showed moderate effects sizes for trauma and depression, with small to no effect for anxiety or dysfunction, respectively. | None. | None. |
| Weobong et al. (2017) | Sustained effectiveness and cost-effectiveness of the Healthy Activity Programme, a brief psychological treatment for depression delivered by lay counsellors in primary care: 12-month follow-up of a randomised controlled trial. | Randomized controlled trial; outcomes at 12 months post intervention. | Healthy Activity Program (HAP): lay counselors conducted 6-8 sessions (behavioral activation as the core psychological framework with added emphasis on strategies such as problem-solving and activation of social networks) vs. enhanced usual care delivered by lay counsellors for 3 months. | n = 493 participants screening more than 14 on PHQ9 in Goa, India. | 10 primary care centers in Goa, India. | HAP participants maintained the gains they showed at the end of treatment through the 12-month follow-up, with lower symptom severity scores than participants who received EUC alone and higher rates of remission. They also fared better on most secondary outcomes, including recovery, any response over time, higher likelihood of reporting a minimal clinically important difference, and lower likelihood of reporting suicidal behaviour. | HAP provides better outcomes at lower costs from a perspective covering publicly funded healthcare services and productivity impacts on patients and their families. | None. |

| **Community Interventions: Mental Health Promotion and Prevention** | | | | |  |  |  |  |
| --- | --- | --- | --- | --- | --- | --- | --- | --- |
| **Author** | **Title** | **Design** | **Intervention** | **Participants** | **Community Setting/Involvement** | **Mental Health Outcomes** | **Individual or Community-level Social Outcomes** | **Other Outcomes** |
| Betancourt et al. (2017) | Family-based promotion of mental health in children affected by HIV: a pilot randomized controlled trial. | Randomized controlled trial; outcomes post intervention and at 3 months. | Family Strengthening Intervention (FSI-HIV), a family home-visiting intervention to promote mental health and improve parent-child relationships in families with caregivers living with HIV. | 82 families (N = 170 children, 48.24% female; N = 123 caregivers, 68.29% female) with at least one HIV-positive caregiver (n = 103, 83.74%) and school-aged child (ages 7–17) (HIV+ n = 21, 12.35%). | Delivered to participants at home in Kayonza District Rwanda. | Group differences observed for children in FSI-HIV with fewer symptoms of depression compared to TAU by both self-report (b = -.246; p = .009) and parent report (b = -.174; p = .035). No significant group differences on conduct problems, functional impairment, family connectedness, or parenting. | None. | None. |
| Buller et al. (2016) | The way to a man's heart is through his stomach?: A mixed methods study on causal mechanisms through which cash and in-kind food transfers decreased intimate partner violence. | Mixed methods, sequential explanatory study, cluster-RCT; outcomes over 6 months. | Six-month food assistance program made of four groups: cash, food, food vouchers, and control. | n = 2,357 households. | Seven urban centers in the northern provinces of Carchi and Sucumbíos consisting of Colombian refugees and low-income Ecuadorian households in Ecuador. | Qualitative and quantitative evidence that the intervention led to reductions in IPV through three pathways operating at the couple, household and individual level: i) reduced day-to-day conflict and stress in the couple; ii) improved family well-being and happiness, iii) increasing women’s decision-making ability through self-confidence and freedom of movement. | Couples on the northern border of Ecuador showed that transfers reduced physical or sexual violence by 30 %. Increased women's decision making, self-confidence and freedom of movement. | None. |
| Cluver et al. (2016) | Can Social Protection Improve Sustainable Development Goals for Adolescent Health? | Longitudinal survey; outcomes at 1 year post intervention. | Social protection in the form of ‘cash’ (economic provision) and ‘care’ (psychosocial support) social protection. | n = 3,515 adolescents (10-18 years) in two South African provinces. | Two urban and two rural health districts in two South African provinces, including all homes with a resident adolescent. | Social protection was associated with significant adolescent risk reductions in SDG 3 (AIDS, tuberculosis, mental health and substance abuse); SDG 5 (sexual exploitation, sexual and reproductive health); and SDG 16 (violence perpetration). | Social protection was associated with significant adolescent risk reductions in SDG 2 (hunger); SDG 4 (educational access). | None. |
| Diaz-Linhart et al. (2016) | Patient Navigation for Mothers with Depression who Have Children in Head Start: A Pilot Study. | Experiment; outcomes over 6 months. | Social work–facilitated patient navigation using lay navigators augmented with engagement interviewing to help mothers with depression engage with mental health care vs. non navigation assistance in accessing care. | n = 47 mothers meeting diagnostic criteria for major depressive episode (MDE) with a child in the Head Start program. | Five Head Start (a U.S. preschool program for low-income children) community based centers using lay navigators in a single metropolitan area, USA. | Not powered to detect group differences. | None. | Feasibility of community-based navigation was established. The study was not powered to detected group-to-group differences although at 6 months more navigated participants engaged with health providers. |
| Doyle et al. (2016). | Can Targeted Intervention Mitigate Early Emotional and Behavioral Problems?: Generating Robust Evidence within Randomized Controlled Trials. | Experiment; outcomes at 24 months. | Preparing for Life (PFL), a 5 year community-based home visiting program (HVP) aiming to improve children’s health and development in disadvantaged communities vs. usual care. | n = 233 pregnant women from one disadvantaged community in Dublin, Ireland. | Community-based home visits in Dublin, Ireland. | The PFL treatment effects for emotional and behavioral difficulties were limited to boys with the greatest level of problems. | None. | None. |
| Ell et al. (2017) | Promotora assisted depression and self-care management among predominantly Latinos with concurrent chronic illness: Safety net care system clinical trial results. | Experiment; outcomes at 6 and 12 months. | Intervention to improve depression and self-care management among patients with diabetes and/or heart disease, the A-Helping-Hand (AHH) intervention in which bilingual promotoras provided 6 weekly psychoeducational sessions followed by boosters vs. Los Angeles County Department of Health Services (LAC-DHS) clinic usual care (UC). | n = 348 Latino (99%) depressed patients (PHQ-9 score ≥10) with comorbid diabetes (85%), heart disease (11%) or both (11%) resident in Los Angeles. | Three Los Angeles County Department of Health Services (LAC-DHS) community clinics, USA. | No group differences in mental health outcomes. | None. | No group differences in quality of life, disease management, and medical care utilization. |
| Gaughran et al. (2017) | Randomised control trial of the effectiveness of an integrated psychosocial health promotion intervention aimed at improving health and reducing substance use in established psychosis (IMPaCT). | Randomized controlled trial; outcomes at 12 and 15 months post intervention. | A Modular Health Promotion Intervention (IMPaCT therapy) vs. treatment as usual (TAU). | Patients with psychosis; community care coordinators in the UK. | 5 UK mental health National Health Service trusts. | None. | Physical and Mental health subscales of Short Form-36 and cardiovascular risk indicators; only 1 significant outcome, reduction of waist circumference in a subsample. | None. |
| Hidrobo et al. (2016) | "The effect of cash, vouchers, and food transfers on intimate partner violence: evidence from a randomized experiment in Northern Ecuador." | Neighborhood cluster randomized control study; outcomes at 6 months. | Six-month food assistance program made of four groups: cash, food transfers, food vouchers, and control. Participants attended monthly nutrition education sessions. | n = 80 neighborhoods and 145 clusters within neighborhoods; n = 2,357 households. | Seven urban centers in the northern provinces of Carchi and Sucumbíos consisting of Colombian refugees and low-income Ecuadorians households in Ecuador. | None. | Food transfers decrease the probability that a woman experiences controlling behaviors, and physical and/or sexual violence by 6 to 7 percentage points or approximately a 19 percent to 30 percent decrease from endline control means. And, there was no evidence  that partners use violence to forcefully extract transfers. | None. |
| Hoffmann et al. (2015) | Developing a Community-Based Tailored Exercise Program for People With Severe and Persistent Mental Illness. | Quasi experiment and description of community based participatory research (CBPR) process. | CBPR and intensive community collaboration to develop and deliver a gender-specific, group-based exercise intervention for people with severe and persistent mental illness (SPMI). | n = 16 individuals with SPMI attending a community human service organization. CBPR comprised CHS leader and staff and researchers from University of Pittsburgh. | Co-designed with community human services (CHS) organization and delivered in its location in a neighborhood in Pittsburgh. | Insufficient power in pilot data to demonstrate significant outcomes. Survey data for mental and physical outcomes showed trends for improved mood, social support, and physical and mental health outcomes. Survey results showed improved trends in mood (positive affective score, p = .67; negative affective score, p = .25), social support (p = .09), and physical health (p = .705) as well as mental health (p = .93) outcomes. The TUG test results showed improved mobility among those that participated regularly (p = .25). | None. | None. |
| Jarman et al. (2016) | Workplace Health Promotion and Mental Health: Three-Year Findings from Partnering Healthy@Work. | Quasi experiment; pretest posttest with outcomes at 3 years. | A comprehensive, multi-component health promotion program, Healthy@Work, targeting modifiable health risks including unhealthy lifestyles and stress. | The health promotion program was delivered to an entire state public service workforce (~28,000 employees) working around the state. A 40% random population sample was taken from the total pool of employees and stratified according to employment condition (permanent, fixed-term/ casual), employment category (full-time, part-time) across the departments. | In workplaces across diverse Tasmanian geography (urban, regional and remote) and state organisations (e.g. health, education, police, forestry, electricity) and occupations in Australia. | The programs in this study did not translate to a change in population mental health. | None. | A small positive association of participation in lifestyle programs with mental health was observed for women but not men. Participation in any mental health and lifestyle program approximately doubled after 3 years. |
| Kilburn et al. (2016) | Effects of a large-scale unconditional cash transfer program on mental health outcomes of young people in Kenya. | Experiment; outcomes over 4 years. | Monthly cash payments of $20 to households that are poor and have at least one orphan or vulnerable child below 18 years of age vs. control. | n = 1,960 households in the Cash Transfer for Orphans and Vulnerable Children (CT-OVC) prgram. | Households in the CT-OVC program in Kenya. | Young men living in households that received unconditional cash transfers were less likely to show depressive symptoms, more likely be hopeful about their lives, and more likely to be healthier than they were previously. The positive impact of the program is stronger among the subgroup of orphans. This study provides evidence that poverty-targeted unconditional cash transfer programs, can improve the mental health of young people in low-income countries. | For young men, residing in an intervention household increased the likelihood of feeling healthier from a year ago. Among young women, the effects of the CT-OVC program were not statistically significant. Finally, intervention effects on being healthy in the past 4 weeks were not statistically significant for young men or young women. | None. |
| Kwon (2015) | Wheel of Wellness Counseling in Community Dwelling, Korean Elders: A Randomized, Controlled Trial. | Randomized controlled trial; outcomes at post 4 week intervention. | Wheel of Wellness counseling, structured, individual counseling based on the Wheel of Wellness model, provided once a week for four weeks vs. no-intervention. | n = 93 of community-dwelling elderly people aged 65 years and over who were cognitively intact and who visited a senior welfare center in Deagu, South Korea. | Delivered in a senior welfare center in Deagu, South Korea. | Significant improvement on depression scores compared to control. Significant improvement on all the wellness lifestyle subtasks except realistic beliefs. Significant improvements in sense of control (t=2.16, p =.033), stress management (t=2.43, p =.017) and perceived wellness (t=2.39, p =.019) compared to the control group. | None. | Significant improvement in nutrition (t=2.44, p =.017), exercise (t=2.10, p =.039), self-care (F=15.31, p <.001) and work (t=2.60, p =.011), compared to the control group. |
| Ljungqvist et al. (2015) | Money and mental illness: a study of the relationship between poverty and serious psychological problems. | Experiment; outcomes at 7 months post intervention. | Treatment and financial support as usual, with an extra allowance of $73 per month vs. treatment as usual. | n = 100 individuals with severe mental illnesses using both psychiatric services and social services. | Regionally-run psychiatric services and municipality-run social services in Sweden. | Significant improvements were found for depression and anxiety, social networks, and sense of self. No differences in functional level were found. | Further studies will be necessary to deepen the knowledge of the relationship between people’s mental problems and financial situation. | None. |
| Mercado et al. (2016) | Generalizability of the NAMI Family-to-Family Education Program: Evidence From an Efficacy Study. | Quantitative study; outcomes at 3 months. | FTF is a 12-week, in-person, curriculum-based program. FTF is a program for families of adults experiencing mental illness. Participants learn about mental illness, treatment models, medication adherence, and the process of rehabilitation in two to three hour dyadic group sessions. | n = 83 people with a family member or as a significant other or friend of an adult with mental illness. (63% were parents of adults with mental illness). | National Alliance on Mental Illness (NAMI) trained family member volunteers in New York City. | None. | Improvements in empowerment, coping, family functioning, self-care, and knowledge, as well as reductions in subjective burden, are consistent with previous FTF studies. Scores on a validated measure of self-care also improved. | This evaluation provides further support for the benefits of FTF and its utility in helping families help themselves and support their relative with a mental disorder. |
| O'Hara et al.. (2017) | Developing a peer-based healthy lifestyle program for people with serious mental illness in supportive housing. | Quasi experiment (single group open trial pre and post test interviews) and experiment with outcomes at 6 and 12 weeks. Qualitative analysis of feasibility conducted. | 2 trials conducted of the intervention, a Peer-based Group Lifestyle Balance (PGLB), a healthy lifestyle program designed to be delivered in community settings (e.g., primary care clinics, community centers) with iterative adaptations made throughout the study to fit the model to needs of people with SMI living in supportive housing. Single group open trial of the intervention conducted then a second trial of intervention vs. 12-week waitlist for the intervention. | n = 7 (no control or comparator) and n= 12 adult residents of a supportive housing agency with a diagnosis of SMI and BMI > 25. | Delivered in a supportive housing agency in New York City serving adults with SMI and using a Housing First model. Peer specialists and social workers co-facilitated the sessions in the supported housing setting. | None. | None. | Intervention adaptations were described e.g.adding peer-specialists as co-facilitators, increasing individualized support and developing strategies to address socioeconomic barriers impacting participants’ ability to engage in healthy lifestyle changes. Study findings suggested that participants with SMI in supportive housing perceived PGLB as feasible and acceptable. |
| Oesterle et al. (2015) | Effects of Communities That Care on Males' and Females' Drug Use and Delinquency 9 Years After Baseline in a Community-Randomized Trial. | Community-randomized trial; outcomes at 9 years. | Communities That Care (CTC), a community prevention capacity building and planning system that organizes and trains coalitions to assess and prioritize young people’s risk, protective factors, and behavioral health problems; and match priorities with tested, effective preventive interventions vs. control (communities given youth surveys but no funding or training from the study). | n = 24 matched communities/ towns in seven states in the USA. Data for this study came from the Community Youth Development Study (CYDS) (Hawkins et al. 2008b), a community-randomized trial of CTC. | Community settings in 7 USA States. | There was no significant overall effect of CTC in the full sample on the mental health secondary outcomes, major depression, and suicidality. | None. | Significant overall effect across prevalence of lifetime and current substance use and delinquency and lifetime abstinence from cigarette smoking (ARR = 1.22) for males only. CTC did not have a sustained effect on current substance use and delinquency nor did it improve the secondary outcomes at age 19 for either gender. |
| Oesterle et al. (2018) | Long-Term Effects of the Communities That Care Trial on Substance Use, Antisocial Behavior, and Violence Through Age 21 Years. | Community-Randomized trial; outcomes at 11 years. | Communities That Care (CTC) prevention system, implemented in early adolescence to promote positive youth development and reduce health-risking behavior vs. control (communities given youth surveys but no funding or training from the study). | n = 24 matched communities/ towns in seven states in the USA; 4407 participants (grade 5 through age 21 years). Data for this study came from the Community Youth Development Study (CYDS) (Hawkins et al. 2008b), a community-randomized trial of CTC. | Community settings in Colorado, Illinois, Kansas, Maine, Oregon, Utah, and Washington State. | There was no significant overall effect of CTC on mental health secondary outcomes, including, major depression, and suicidality. | The CTC system increased the likelihood of sustained abstinence from gateway drug use by 49% and antisocial behavior by 18%, and reduced lifetime incidence of violence by 11% through age 21 years. In male participants, the CTC system also increased the likelihood of sustained abstinence from tobacco use by 30% and marijuana use by 24%, and reduced lifetime incidence of inhalant use by 18%. | This study provides evidence that the CTC is an effective approach to improving public health in the long term by preventing the incidence of health-risking behaviors many years after the most direct exposure to evidence-based programs and policies. |
| Ran et al. (2015) | The effectiveness of psychoeducational family intervention for patients with schizophrenia in a 14-year follow-up study in a Chinese rural area. | Experiment; outcomes at 14 years. | 9 month psychoeducational family intervention for families experiencing schizophrenia. | n = 326 patients with schizophrenia and their informants who participated in a 1994 trial of a psychoeducational intervention. Among all subjects alive (n = 245) in 2008 at 14 year follow up, 238 subjects (97.1%) finished the follow-up evaluations. | Community settings in rural Xinjin County, China as part of the Chengdu Mental Health Project (CMHP). | Suggested ongoing 14 year effects on treatment adherance and work ability. No significant difference at 14 years in mental illness symptoms. | None. | No difference in marital status outcome. |
| Roopnarine et al. (2017) | The moderating role of relationship skills education on depressive symptoms in fathers with young children. | Experiment; Outcomes at 21 months. (Data used from the baseline child age 15 month assessment and follow up age 36-month assessments of the Building Strong Families Study (BSF)). | Relationship skills education programs that had individual support from family coordinators, assessment and referral to support services and delivered in group settings vs. delayed waiting list for relationship skills education program. | n = 2,540 low-income fathers who reported at least one depressive symptom with pre school age children enrolled in the Building Strong Families (BSF) Study, USA. | 8 diverse sites in the USA delivering the BSF study. | Relationship skills education classes that focused on trust, intimacy, and communication led to a decrease in depressive symptoms in fathers with preschool-aged children. | None. | Predictors of attendance at relationship skills education included relationship status, economic stability, and age of the father. |
| Sajatovic et al. (2016) | Targeted Self-Management of Epilepsy and Mental Illness for individuals with epilepsy and psychiatric comorbidity. | Experiment; outcomes at 12 and 16 weeks. | A novel Targeted Self-Management intervention for Epilepsy (E) and Mental Illness (MI) (TIME) in individuals with co-morbid epilepsy and mental illness vrs treatment as usual (TAU). TIME uses peer educators with E-MI to model self-management. | n = 44 adults, mean age 48.25 (SD = 11.82) with co-morbid diagnoses of a severe and persistant mental disorder and epilepsy. | Group intervention delivered in accessible community location (exact venues not specified) and phone intervention delivered in subjects homes. Peer educators involved in intervention delivery. | Significant reduction in depressive symptoms (assessed by the Montgomery Asberg Depression Rating Scale (MADRS)) in intervention vrs control. No significant group differences observed in other mental health related outcomes (global psychiatric symptomseverity, quality of life, social support, and self-efficacy ). | None. | Intervention was feasible to deliver and acceptable to subjects. |
| Skeffington et al. (2016) | The Primary Prevention of PTSD in Firefighters: Preliminary Results of an RCT with 12-Month Follow-Up. | Randomized control trial; outcomes at 6 and 12 months. | A PTSD primary prevention training program, Mental Agility and Psychological Strength (MAPS) included in firefighters recruit training school curriculum vs. “Training as Usual” (TAU). The MAPS program focusses on building knowledge of psychological wellbeing and PTSD as well as practical skills such as cognitive re-structuring, support seeking, and self-soothing or self-moderating. | n = 77 (73 male and 4 female) Trainee Firefighters (TFFs) at the DFES Training Academy in Perth, not currently meeting critera for any mental health condition but at risk of PTSD because of their profession. | Within training school setting for recruits in Department of Fire and Emergency Services (DFES) Western Australia. | No group differences in mental health outcomes including primary prevention of mental health issues, impact on social support or coping strategies. | None. | Group difference in trauma knowledge associated with intervention. |
| Toohey et al. (2016) | Caregiver Positive and Negative Appraisals: Effects of the National Alliance on Mental Illness Family-to-Family Intervention. | Experiment; outcomes post intervention at 3 months. | The Family-to-Family intervention (FTF), a free, 12 week peer-run community-based, education and support program offered nationally by the National Alliance on Mental Illness (NAMI) to any family member, partner, or close friend of an adult with mental illness vs. 3 month waiting list for intervention. | N = 318 family members of individuals with mental illness. | Within community settings in Maryland USA. | None. | None. | The FTF program increased caregiver endorsement of positive caregiving appraisals but did not reduce their endorsement of negative caregiving appraisals, compared to controls. |
| Wansink et al. (2015) | Effects of preventive family service coordination for parents with mental illnesses and their children, a RCT. | Randomized control trial; outcomes at 9 and 18 months. | The Preventive Basic Care Management (PBCM) program (based on the broker model of case management and designed to organize and coordinate psychiatric and preventive services for families using a family-focused strength-oriented rehabilitation model) vs. treatment as usual with written information and signposting to services. | n = 99 outpatients of a community mental health center who were a caregiver of a child between age 3 and 10 years and who had long-standing psychiatric problems, parenting problems, and an accumulation of risk factors for poor parenting. | In community mental health settings in the urbanized western part of the Netherlands. | No group differences in mental health outcomes of parenting stress. No effects on prevention of child behavioral problems. | None. | Authors concluded intervention was feasible. Small to medium significant positive effect on parenting skills. |
